# Materials informatics approach using domain modelling for exploring structure–property relationships of polymers

**DOI:** 10.1038/s41598-022-14394-5

**Published:** 2022-06-22

**Authors:** Koki Hara, Shunji Yamada, Atsushi Kurotani, Eisuke Chikayama, Jun Kikuchi

**Affiliations:** 1 Graduate School of Medical Life Science, Yokohama City University, 1-7-29 Suehiro-cho, Tsurumi-ku, Yokohama, Kanagawa 230-0045 Japan; 2 RIKEN Center for Sustainable Resource Science, 1-7-22, Tsurumi-ku, Yokohama, 230-0045 Japan; 3 Department of Information Systems, Niigata University of International and Information Studies, 3-1-1 Mizukino, Nishi-ku, Niigata-shi, Niigata 950-2292 Japan; 4 Graduate School of Bioagricultural Sciences, Nagoya University, 1 Furo-cho, Chikusa-ku, Nagoya, Aichi 464-0810 Japan

**Keywords:** Solid-state NMR, NMR spectroscopy

## Abstract

In the development of polymer materials, it is an important issue to explore the complex relationships between domain structure and physical properties. In the domain structure analysis of polymer materials, ^1^H-static solid-state NMR (ssNMR) spectra can provide information on mobile, rigid, and intermediate domains. But estimation of domain structure from its analysis is difficult due to the wide overlap of spectra from multiple domains. Therefore, we have developed a materials informatics approach that combines the domain modeling (http://dmar.riken.jp/matrigica/) and the integrated analysis of meta-information (the elements, functional groups, additives, and physical properties) in polymer materials. Firstly, the ^1^H-static ssNMR data of 120 polymer materials were subjected to a short-time Fourier transform to obtain frequency, intensity, and *T*_2_ relaxation time for domains with different mobility. The average *T*_2_ relaxation time of each domain is 0.96 ms for Mobile, 0.55 ms for Intermediate (Mobile), 0.32 ms for Intermediate (Rigid), and 0.11 ms for Rigid. Secondly, the estimated domain proportions were integrated with meta-information such as elements, functional group and thermophysical properties and was analyzed using a self-organization map and market basket analysis. This proposed method can contribute to explore structure–property relationships of polymer materials with multiple domains.

## Introduction

Traditional design approaches for materials are experimentally driven, facing significant challenges due to the vast design space of materials. Experimental science can be supported by materials informatics^1–3^ that makes full use of theoretical and computational science such as density functional theory (DFT)^4,5^ and molecular dynamics (MD)^6^ and data science using computers (Artificial Intelligence; AI)^7,8^. Computational science solves equations numerically based on theory and physical models. On the other hand, data science explores candidate materials with a certain function from a large quantity of material data. Its physical meaning needs to be verified by experimental, theoretical and computational science. The meta-information involved in the production process of the material also plays an important role in desired materials development^9,10^.

In recent years, the development of sustainable polymers that meet the needs of consumers without destroying the environment has become an important issue due to global problems such as marine pollution, waste disposal, and global warming caused by plastics^11,12^. Furthermore, “carbon–neutral” bio-based polymers, such as polylactic acid (PLA), polybutylene succinate (PBS), poly(3-hydroxybutyrate-co-3-hydroxyhexanoate) (PHBH), and poly-ε-caprolactone (PCL), have become a focus in the era of biorefinery materials as an alternative to oil-based materials^13,14^. Polymers such as PLA^15^, PCL^16^, are multiple domain systems, are often employed as high-performance materials which can display various properties.

Solid-state nuclear magnetic resonance (ssNMR) spectroscopy is a powerful tool that is used to characterize the native structure, components and dynamics of solid-state samples at the atomic level, and has been increasingly applied in material sciences^17,18^. In addition, NMR measurements, especially low magnetic field NMR, is a method for routine material evaluations, which has produced a lot of NMR datasets^19^. Typical ssNMR methods are cross-polarization (CP)/magic-angle spinning (MAS) methods with elimination of linewidth broadening due to chemical shift anisotropy for high resolution. On the other hand, there are different complementary approaches to tackle complexity of polymer domain structure. ^1^H-static ssNMR can be applied to quantify domain mobility in terms of dynamic heterogeneity^20^. In addition, the use of magic-and-polarization echo (MAPE)^21^ and double-quantum (DQ)^22^ filters can determine the spectral parameters for the mobile amorphous domains with the long-time decay and the strongly dipole–dipole-coupled crystalline domains with the quickly decay, respectively.

In the case of characterization of a solid-state sample with domains of rigid, intermediate and mobile types, the ^1^H-static ssNMR measurement is useful as a measure of the kinetic nature of higher order structures, although its analysis is difficult because the spectrum is broadened and overlapped^23^. Therefore, application of signal deconvolution is needed to characterize structure and property of the sample. Several methods for spectral separation^19^, fitting and numerical simulation^24^ such as SIMPSON^25^, SPINEVOLUTION^26^, dmfit^27^, EASY-GOING deconvolution^28^, INFOS^29^, Fityk^30^, ssNake^31^, and a noise reduction method based on principal component analysis^32^ have been developed. So far, in NMR data analysis, signal simulation and fitting have targeted only the frequency domain or the time domain. In our previous study, we proposed signal deconvolution methods that combines short-time Fourier transform (STFT; a time–frequency analytical method) and probabilistic sparse matrix factorization^33^, and non-negative tensor/matrix factorization^34^. In our method using STFT, by simulating the signal for both the frequency and time domain, it was possible to separate the signal related to the motility characteristics of the domain structure based on the indicators of chemical shift and *T*_2_ relaxation time. The NMR signal can be calculated by functions such as Lorentzian^35^, Gaussian^36^, and Voigt^37^ in the frequency domain, and by the *T*_2_ relaxation equation^38^ in the time domain. In addition, the difference in *T*_2_ relaxation times can be adjusted by the Weibull coefficient^39^. Analysis of the relaxation time of a sample's free-induction decay (FID) provides important insights into the chemical composition, structure, and mobility of the sample^38,40^.

The polymer domain structure has a significant influence on their macroscopic properties^41^. Materials informatics, which is the emerging field, support analysis of relationships of structure and property from materials data sets^2,3,7,8,42^. NMR signal has potential for use as a descriptor having the structural features of the molecules contributing to their physical/chemical/biological properties^43^. In previous studies, a self-organization map (SOM) has been applied to tool wear monitoring^44^. Market basket analysis (MBA) has been applied to predict drug-drug interactions^45^. Bayesian optimization has been applied in real and virtual degradable experiments of bioplastics^46^. Generative topographic mapping regression (GTMR) has also been applied to the analysis of CP-MAS spectra to predict ^13^C NMR spectrum of the material in its solid-state based on its thermophysical properties^34^. Machine learning methods have applied for various material studies such as cloud-point engineering of polymers^47^, prediction of drug-polymer amorphous solid dispersion miscibility and stability^48^, atomic/inter-atomic properties prediction^49^, solubility prediction^50^, descriptor selection for investigating physical properties of biopolymers in hairs^51^, classification of the membrane materials^52^, prediction of crystallization tendency^53^, prediction of density, glass transition temperature, melting temperature, and dielectric constants of polymer^9^, macromolecular modeling^54^.

In this study, we propose a materials informatics approach to explore the structure–property relationships of polymers that combines the polymer domain modeling and the integrated analysis of polymer materials meta-information. For polymer domain modeling, ^1^H-static ssNMR spectral parameters obtained using STFT were utilized, including *T*_2_ relaxation time, frequency, and intensity. The domain structure with different mobility in the polymeric material was estimated by fitting the physical indices such as *T*_2_ relaxation time, frequency, and linewidth. In addition, using a SOM and MBA, the relationships between the estimated domain structure and the meta-information such as elements, functional group, and thermophysical property were explored.

## Results and discussion

### A materials informatics approach to exploring structure–property relationships using domain modeling

**Figure 1:**
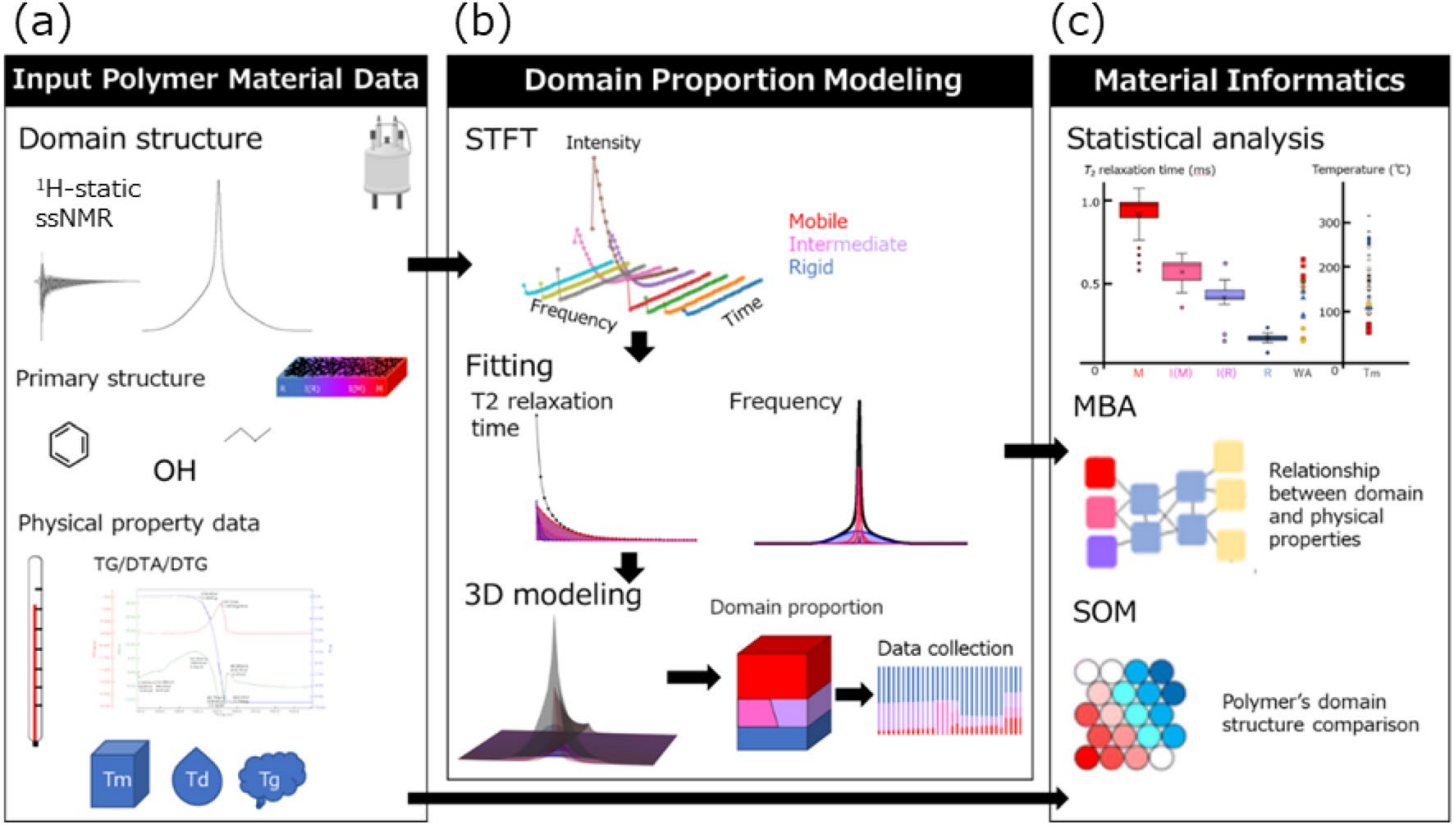
Conceptual diagram of materials informatics approach to exploring structure–property relationships using domain modeling. (**a**) To exploring relationships between structure and property of polymer materials, ^1^H-static solid-state NMR (ssNMR) spectrum, primary structure information and physical property data obtained from TG, DTA and DTG were used. (**b**) Domain modeling was applied to the input ^1^H-static ssNMR spectrum of polymer materials to obtain domain structural information. (**c**) The domain proportion, primary structure data and thermophysical property data were integrated for data analysis such as statistical analysis, SOM and MBA. ^1^H-static ssNMR, Proton static solid-state nuclear magnetic resonance; TG, Thermogravimetry; DTA, Differential thermal analysis; DTG, Derivative thermogravimetry; T_m_, Melting temperature; T_d_, Thermal decomposition temperature; T_g_, Glass transition temperature; STFT, Short-time Fourier transform; MBA, Market basket analysis; SOM, Self-organization map.

The conceptual diagram of materials informatics approach to exploring structure–property relationships using domain modeling is shown in the Fig. 1. The detailed analytical flow of this method is shown in Supporting Information Fig. S1. We have utilized the input polymer information that are ^1^H-static ssNMR (^1^H-static ssNMR) data, primary structure of the polymer, and thermophysical property data (TG/DTA/DTG). Then, frequency and time information are obtained by STFT against FIDs obtained by ^1^H-static ssNMR. The domain modeling method is the following. The domain components are firstly separated by fitting the obtained frequency and time information (Fig. S2). Secondly, the domain component ratio is calculated by 3D modeling (Fig. S3). To reduce the error between total of the simulated values of domain components using our signal processing method and the original static data, Bayesian optimization as one of the optimization methods was performed using Eq. 1 (see in the Materials Methods section) in the text to search for the ratio between the mobile and rigid components according to Eqs. (2–5). After that, we performed statistical analysis, MBA, and SOM, which are materials informatics methods, on the obtained domain information, primary structure information, and thermophysical property information to associate the structure and physical properties. The detailed results are shown in the following sections.

### Results of domain modeling of polymer materials using time–frequency simulation of ^1^H-static ssNMR spectra

**Figure 2:**
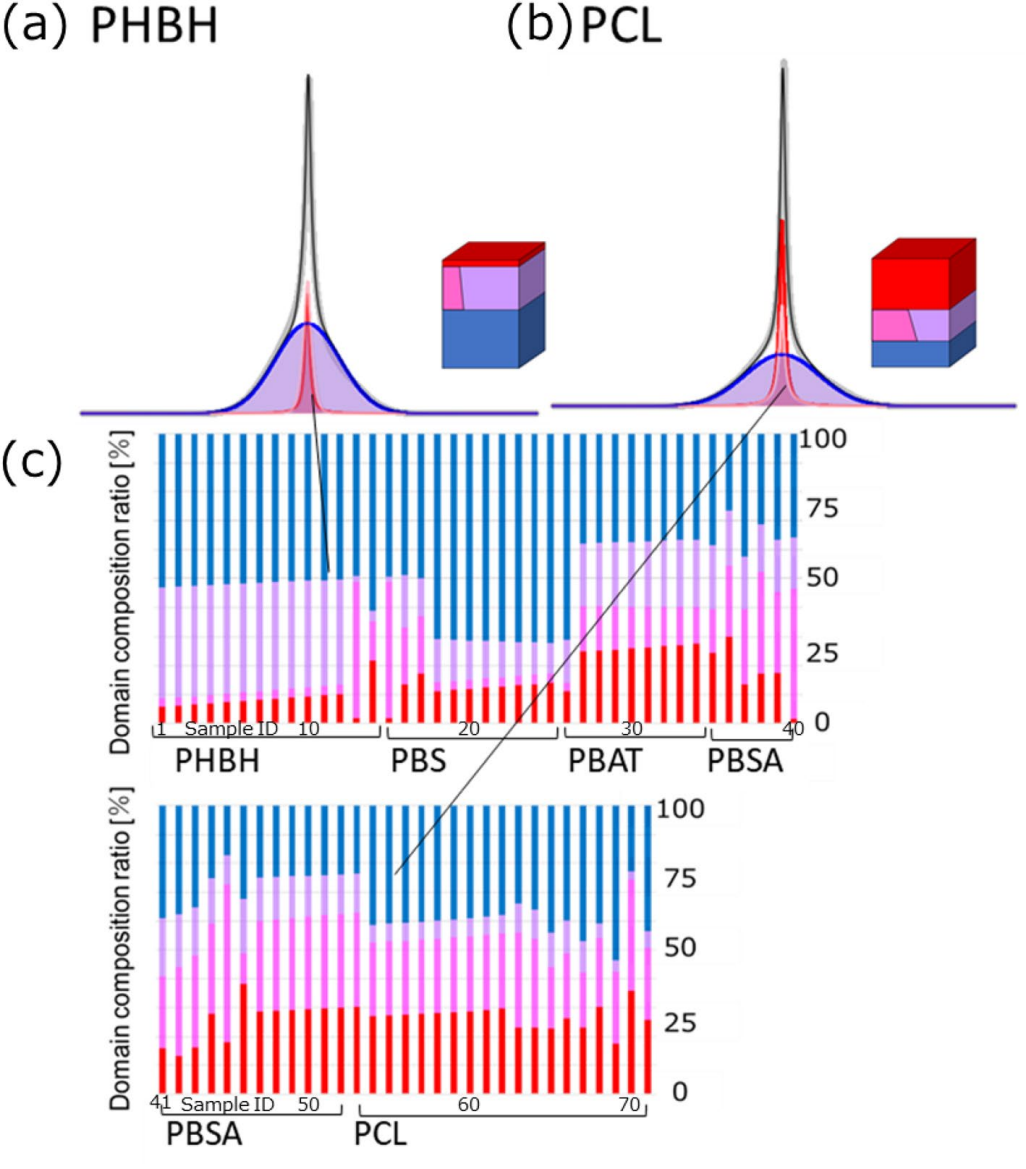
Results of domain proportion calculation of polymer materials. The ^1^H-static ssNMR spectra of 71 polymer materials were separated by domain modeling. Based on the results, the domain models are shown. (**a**) An example of PHBH, (**b**) an example of PCL, and (**c**) the domain ratios of 71 polymer materials. Polymer sample ID numbers in the figure refer to Table S2. Red: Mobile, Magenta: Intermediate (Mobile), Violet: Intermediate (Rigid), Blue: Rigid.

The ratios of the domain components were calculated from the volume ratios obtained from domain modeling of 71 samples of the polymer materials (Fig. 2). Here, the domain component is defined as a region of higher-order structure distinguished by molecular mobility in polymers. NMR characterizes this domain component by analyzing the spectral widths or *T*_2_ relaxation time. The volumes of four domain component (Mobile, Intermediate (Mobile), Intermediate (Rigid) and Rigid) are calculated from Eqs. 2–5 ($M_{Mobile}$, $M_{IM}$, $M_{IR}$ and $M_{Rigid}$). The volume ratio is defined as the ratio of the volume of each domain component estimated by 3D simulation based on those equations to total volume. We have classified analyzed data in this study into four domain components: Mobile, Intermediate (Mobile), Intermediate (Rigid), and Rigid domain components, those we regard to mobile, slightly mobile, slightly rigid, and rigid components in their material states, respectively. As a result, the domain component ratios indicated differences among not only different polymer materials but also similar ones that composed of the same monomers, which can be attributed to the molding conditions and molecular weight. In the case of PCL (Fig. 2, upper right), which is the sample with the most Mobile domain component: the Mobile domain component ratio was 37.7%; the Intermediate (Mobile) domain component ratio was 11.2%; the Intermediate (Rigid) domain component ratio was 18.1%; and the Rigid domain component ratio was 33.0%. In the case of PHBH sample (Fig. 2, upper left), which has the highest Rigid domain component: the Mobile domain component ratio was 10.4%; the Intermediate (Mobile) domain component ratio was 3.1%; the Intermediate (Rigid) domain component ratio was 36.5%; and the Rigid domain component ratio was 50.5%. From this result, we were able to calculate the difference in domain ratios caused by the different monomers used to synthesize the polymer.

**Figure 3:**
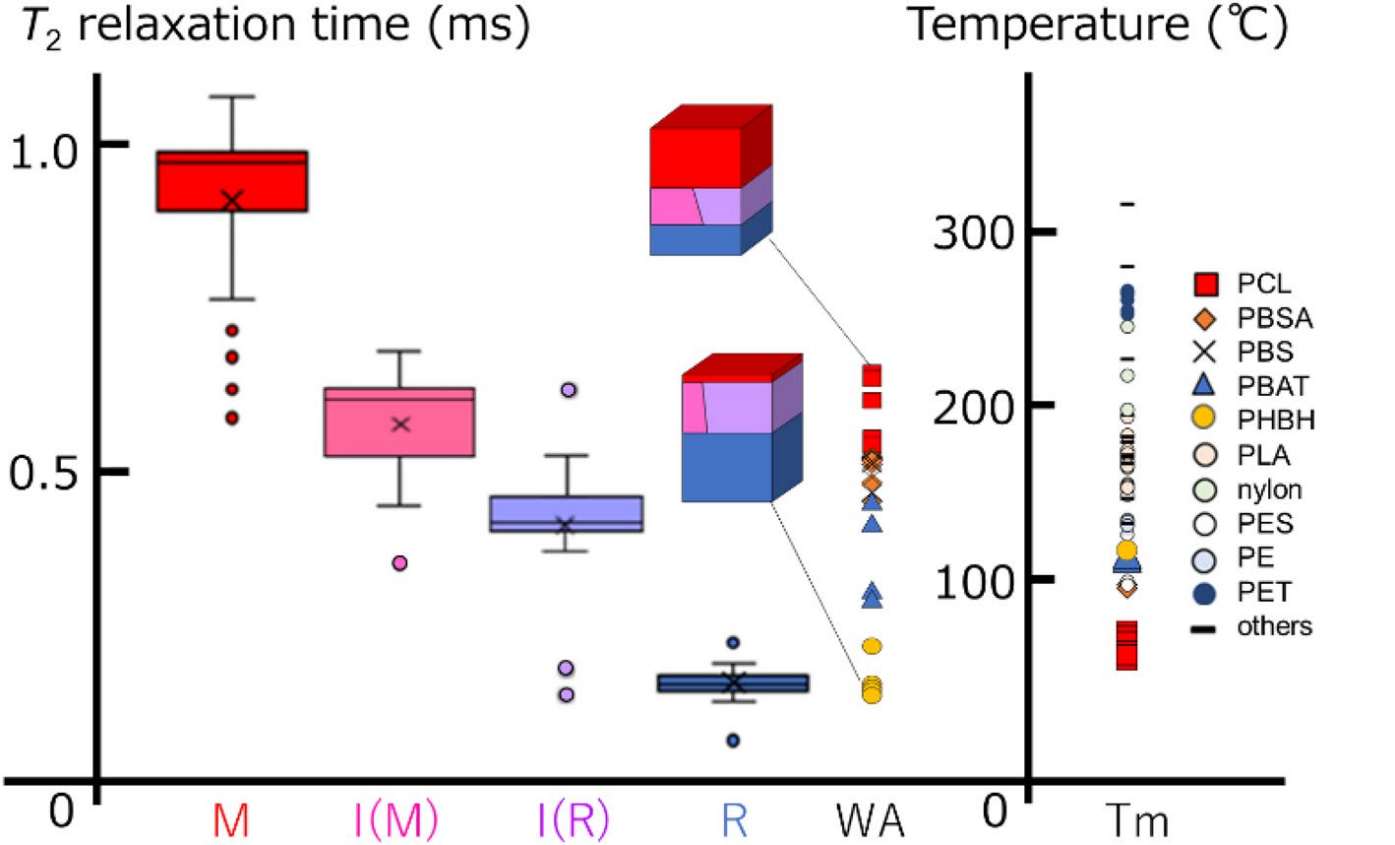
Diagram of polymer properties with *T*_2_ relaxation time among four domain components and melting temperature. M: Red box plot; Mobile, I(M): Pink box plot; Intermediate (Mobile), I(R): Violet box plot; Intermediate (Rigid), R: Blue box plot; Rigid, WA: Weighted average, *T*_m_: Melting temperature. x: Average *T*_2_ relaxation time. The box and whisker plot displays the minimum, first quartile, median, third quartile, maximum, and outliers. The estimated domain proportion models were inserted for the highest PCL and lowest PHBH of WA.

The calculated distributions of *T*_2_ relaxation time and thermophysical properties of the domain components of each polymer are shown (Fig. 3). The box-and-whisker plot shows that the average *T*_2_ relaxation time information is 0.96 ms for Mobile, 0.55 ms for Intermediate (Mobile), 0.32 ms for Intermediate (Rigid), and 0.11 ms for Rigid. The weighted average (WA) of the polymer materials showed a distribution among the samples, among which PCL (Fig. 3, red squares) was high and PHBH (Fig. 3, yellow circles) was low. The same was true for the results of thermal analysis spectral data (Table S3). Based on the domain component ratios, the estimated domain ratio diagrams were inserted for the highest PCL and lowest PHBH of WA.

### Self-organizing map analysis integrating domain proportions and quantitative spectral data in polymer materials

**Figure 4:**
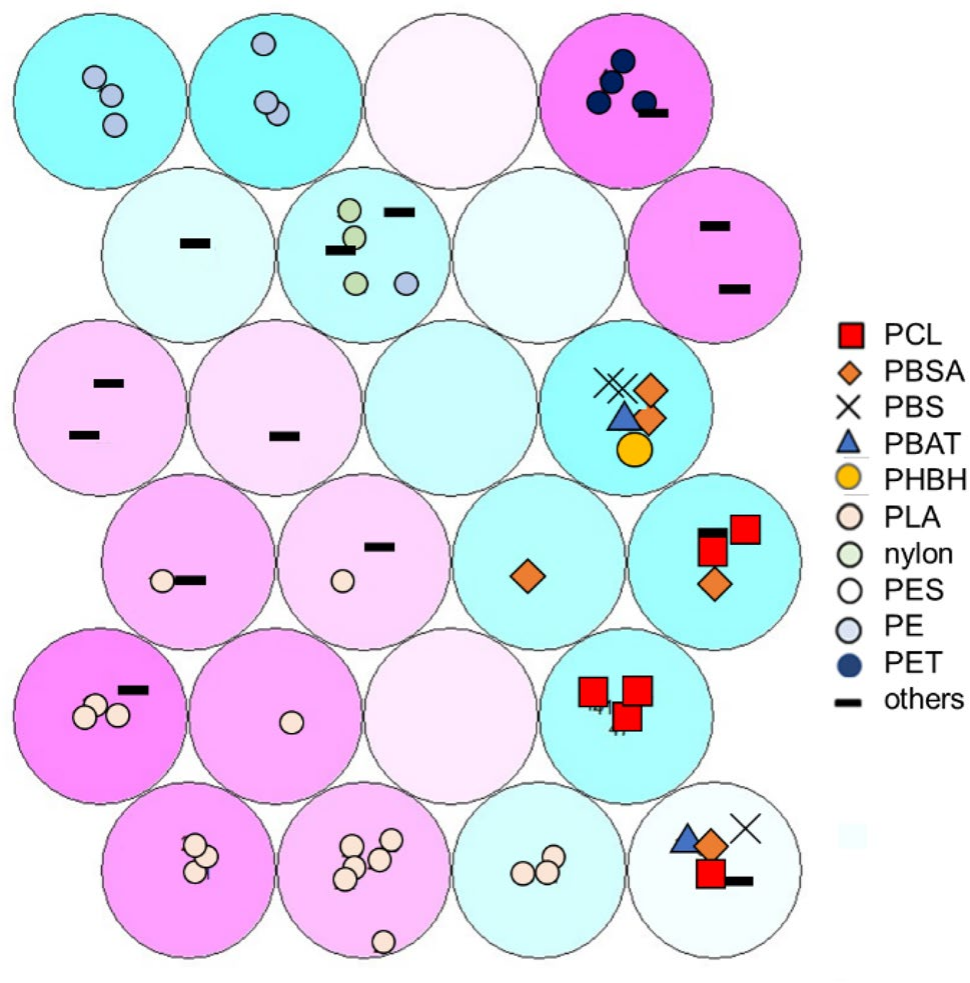
Result of SOM. Visualization of 3D SOM compressed into 2D SOM with 4 × 6 segments, red: positive, blue: negative. red square: PCL, orange: PBSA, black cross: PBS, blue triangle: PBAT, yellow circle: PHBH, light pink: PLA, light green: nylon, white: PES, light blue: PE, navy blue: PET, black bar: other polymers. Polymer numbers in the figure refer to Table S1.

In order to evaluate the relationships between domain structure and thermophysical properties of polymer materials, we integrated the domain proportion described in the previous section, ^13^C-CP/MAS spectra^55^, which easily reflect primary chemical structure, and quantitative thermophysical data (thermogravimetry (TG), differential thermal analysis (DTA), derivative thermogravimetry (DTG), differential scanning calorimetry (DSC))^56^. To capture the characteristics of the integrated data, clustering by SOM was performed (Fig. 4). For input data of SOM, the domain component information calculated by the component separation method and meta-information of thermophysical properties, elements, and functional groups used is listed in Tables S1 and S2. These materials clustered in the following way i.e., navy blue circle symbols are polyethylene terephthalate (PET), light blue circle symbols are polyethylene (PE), and the clusters of these polymer materials are on the top, blue triangle symbols are poly(butylene adipate-co-terephthalate) (PBAT), light pink circle symbols are polylactic acid (PLA), light green circle symbols are poly(hexano-6-lactam) (nylon), and the clusters of these polymer materials are on the bottom left, black cross symbols are polybutylene succinate (PBS), orange diamond symbols are poly(butylene succinate-co-butylene adipate) (PBSA), and red square symbols are PCL, and the clusters of these polymer materials are found to exist solidly in the lower right corner, respectively. These clusters formed two groups: high heat resistant polymers with many rigid domain components, and low heat resistant polymers with many mobile domain components^57,58^. The results of thermal analysis for PET, PE, PBAT, PLA, nylon, PBS, PBSA, and PCL were consistent with their characteristics shown above (Fig. S4, Table S3).

### Market basket analysis integrating quantitative domain proportion and qualitative meta-information in polymer materials

**Figure 5:**
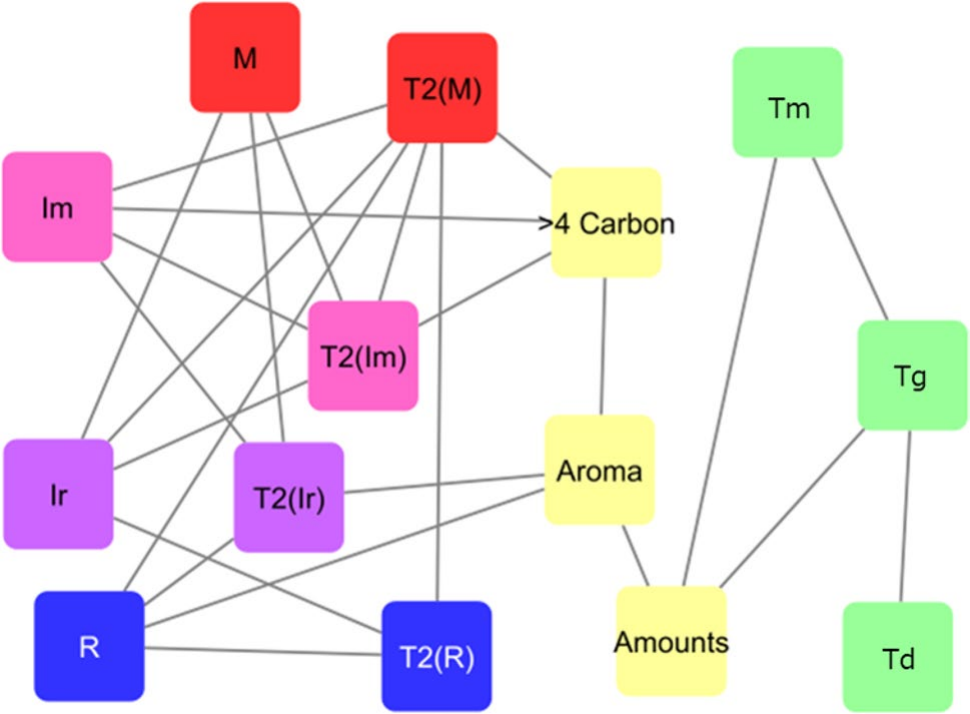
Selected MBA network for *T*_2_ relaxation time data. From the results of the market basket analysis, only the relationships between the simulated domain structure information and *T*_2_ relaxation time data, experimental melting temperature (T_m_), thermal decomposition temperature (T_d_), glass transition temperature (T_g_), and data on primary structure were extracted. M: Mobile domain, Im: MAPE Filter-derived Intermediate domain (Intermediate (Mobile)), Ir: DQ Filter-derived Intermediate domain (Intermediate (Rigid)), R: Rigid domain, T2(M): *T*_2_ relaxation time information for mobile domain, T2(Im): *T*_2_ relaxation time information for Intermediate (Mobile) domain, T2(R): *T*_2_ relaxation time information for rigid domain, T2(Ir): *T*_2_ relaxation time information for Intermediate (Rigid) domain, > 4 carbon: linearly connected methylenes > 4 carbons, Aroma: aromaticity, Amounts: sample amounts.

The MBA was performed to evaluate the relationships between the quantitative domain proportion as well as qualitative meta-information such as elements (linearly connected methylenes > 4 carbons), functional groups (aromaticity), and thermophysical properties. For input data of MBA, the domain component information calculated by the component separation method and meta-information of thermophysical properties, elements, and functional groups used is listed in Tables S1, S2 and S3. Using the transaction data based on the MBA, a network diagram is shown in Fig. 5, where the *T*_2_ information of the four domain components (Mobile, Intermediate (Mobile), Intermediate (Rigid), and Rigid) shows a high lift value with the primary structure information (aromaticity, linearly connected methylenes > 4 carbons). While the thermophysical properties (melting temperature, *T*_m_; thermal decomposition temperature, *T*_d_; glass transition temperature, *T*_g_) show a dominant lift value with their structure information. The lift value here is one of the indicators for correlation analysis in MBA. Figure S5 shows the MBA network diagram using the temperature information from thermal analysis, where the Mobile domain ratio correlated with the lower temperature (< 100 °C) of thermophysical properties. The Intermediate (Mobile) domain ratio from the MAPE Filter and the Intermediate (Rigid) domain ratio from the DQ Filter showed intermediate thermophysical properties (100 to 300 °C). The rigid domain ratio correlated with the lower temperature (< 100 °C) and of the higher temperature (> 300 °C) thermophysical properties. In common, PCL is a thermoplastic biodegradable polyester with good thermal processability and low melting point^57^. In PCL, there is a melting temperature at 66.5 °C of DTG (Fig. S4, Table S3). While the DTG peak of lower temperature is correlated with the Rigid domain (Fig. S5a).

## Conclusion

In the development of materials, in addition to the chemical structure from the primary to the higher-order, meta-information such as molding process and additives are important factors because these have great influences on the final material properties. We have developed a materials informatics approach that combines the domain modeling and the integrated analysis of materials meta-information. To estimate the domain structure information, we have introduced a time–frequency simulation method for calculating multiple domain components from ^1^H-static ssNMR spectra. In our integrated analysis of domain proportions and meta-information, SOM was a useful tool for capturing trends across polymer material data. On the other hand, MBA was able to investigate the strong relationships between structure and meta-information in individual materials, including qualitative data as well as quantitative data. The relationships between mobility of domain structure and melting temperature were similar to the results shown by SOM and MBA (Figs. 3, 4, 5)^57,59^. This materials informatics approach is expected to efficiently explore relationships between structure and properties of high-performance and low environmental impact polymer materials.

## Materials and methods

### Materials

Polymer materials (Table S1) were prepared using a press molding machine (H300-01, AS ONE Corp., Osaka, Japan) and molding methods reported in a previous study^46^.

### Time–frequency simulation of ^1^H-static ssNMR spectra

The time–frequency simulation method was developed in Python 3, by using the packages of nmrglue^60^ for processing of NMR data, Scipy.signal for the Fourier transform, STFT, and mathematical processing, the curve-fit function of scipy.optimize and BayesianOptimization for the fitting process, and mpl_toolkits for visualization of 3D (time, frequency, and intensity) simulation model. Before applying this method, the FID data was phase-corrected, baseline-corrected, and inverse Fourier transformed using TopSpin (Bruker-BioSpin, MA, USA). In order to calculate the ratio between the Mobile and Intermediate (Mobile) domain components obtained from the MAPE filtered spectra, and the Rigid and Intermediate (Rigid) domain components obtained from the DQ filtered spectra, the calculation errors between the four domain components and the STFT ^1^H-static spectra (Static) were calculated using the following equation (Eq. 1).

1 $$Calculation\;error = Static - \left( {\alpha \times \left( {M_{Mobile} + M_{IM} } \right) + \beta \times \left( {M_{IR} + M_{Rigid} } \right)} \right)$$

After finding the α and β parameters that minimize the error, we created a 3D model of the four domain components based on the frequency and *T*_2_ relaxation time information.

The domain component ratios contained in the polymer material were calculated using the following equation (Eq. 2–5).

2 $$M_{Mobile} = \frac{M\left( t \right)L\left( x \right)}{{M_{0}^{\prime } }}$$

3 $$M_{IM} = \frac{M\left( t \right)IM\left( x \right)}{{M_{0}^{\prime } }}$$

4 $$M_{IR} = \frac{M\left( t \right)IR\left( x \right)}{{M_{0}^{\prime } }}$$

5 $$M_{Rigid} = \frac{M\left( t \right)G\left( x \right)}{{M_{0}^{\prime } }}$$

A detailed description of the time–frequency simulation method is given in Supporting Information Figs. S1 and S2.

The weighted average (WA) of the *T*_2_ relaxation times for a single polymer material was calculated using the following equation (Eq. 6).

6 $$\begin{aligned} WA & = \left\{ {M_{Mobile} T_{2} \left( {Mobile} \right) + { }M_{IM} T_{2}^{IM} + { }M_{IR} T_{2}^{IR} } \right. \\ & \quad \left. { + \;{ }M_{Rigid} T_{2} \left( {Rigid} \right)} \right\}/\left( {M_{{mobile{ }}} + M_{IM} { } + { }M_{IR} + M_{Rigid} } \right) \\ \end{aligned}$$

### Self-organizing maps of domain proportions and thermophysical data in polymer materials

In the integrated analysis of domain proportions and meta-information of polymer materials, a SOM was produced using the R package kohonen^61^. In order to evaluate the relationships between domain structure and thermophysical properties of polymer materials, we integrated the domain component information calculated by the component separation method, ^13^C-CP/MAS spectra, which easily reflect primary chemical structure, and quantitative thermophysical data (TG, DTA, DTG, DSC). To capture the characteristics of the integrated data, clustering by SOM was performed (Tables S1, S2). A basic explanation of SOM is presented in the SOM section of the Supporting Information.

### Market basket analysis of domain proportions and meta-information in polymer materials

MBA was performed using the R package arules^62,63^. Linearly connected methylenes > 4 carbons, oxygen containing and aromaticity was set to 1 if true and 0 if false. The numeric data of domain component information calculated by the component separation method and meta-information of thermophysical properties, elements, and functional groups listed in Tables S1, S2 and S3, were converted to “high” and “low” ranked data. The “high” or “low” ranked data were defined as the top 25% or the bottom 25% of all values. Association rules were determined using criterion values of support, confidence, and lift. Since lift values < 1 do not independent relationship as association rules, this study adopted a cutoff value of 1 as a lift value threshold for association rules. In addition to this, the probabilities of random occurrences are 6.25% for support and 25% for confidence because each variable was ranked by using the top or bottom 25% of all values. The maxlen (maximum size of mined frequent item sets) were set to 2. A basic explanation of MBA is presented in the MBA section of the Supporting Information. The association network was visualized using the Cytoscape program.

### Tool development for automated spectral simulation

We have created a Bayesian optimization-based^50^ spectral simulation tool that automates the *T*_2_ relaxation time domain fitting, frequency fitting, and 3D domain modeling of our domain component separation method. The details of the Python program including *T*_2_ relaxation time information, frequency information, and 3D domain modeling of the present domain component separation method can be obtained at https://github.com/riken-emar/matrigica. For improving the level of accuracy of the prediction, intermediate regression models were employed when performing in-phase machine learning. In addition, we developed a website dedicated to the established domain component ratio calculation, which is freely available at http://dmar.riken.jp/matrigica/.

## Supplementary Information

Supplementary Information.

## Data Availability

The analytical tool and numerical data for association analysis used in this study were deposited on the following websites: http://dmar.riken.jp/NMRinformatics/MatRigiCa.zip and http://dmar.riken.jp/NMRinformatics/DatasetForMatRigiCa.zip.
