# Supplementary material for: Materials informatics approach using domain modelling for exploring structure–property relationships of polymers
Source: Sci Rep. 2022 Jun 22;12:10558. doi: 10.1038/s41598-022-14394-5 (PMC9217937; doi:10.1038/s41598-022-14394-5)
Supplement: Supplementary file 1 — Supplementary Information. [file 41598_2022_14394_MOESM1_ESM.docx]

# Supporting Information for

Materials informatics approach using domain modelling for exploring structure-property relationships of polymers

Koki Hara ^1,2^, Shunji Yamada ^2^, Atsushi Kurotani ^2^, Eisuke Chikayama ^2,3^ and Jun Kikuchi ^1,2,4,^*

^1^ Graduate School of Medical Life Science, Yokohama City University, 1-7-29 Suehiro-cho, Tsurumi-ku, Yokohama, Kanagawa 230-0045, Japan; [koki.hara@a.riken.jp](file:///C:\Users\hkzig\AppData\Local\Packages\microsoft.windowscommunicationsapps_8wekyb3d8bbwe\LocalState\Files\S0\5\Attachments\koki.hara@a.riken.jp)

^2^ RIKEN Center for Sustainable Resource Science, 1-7-22, Tsurumi-ku, Yokohama, 230-0045, Japan; [shunji.yamada@riken.jp](file:///C:\Users\junki\AppData\Local\Microsoft\Windows\INetCache\Content.Outlook\TJ0464QP\shunji.yamada@riken.jp), [atsushi.kurotani@riken.jp](file:///C:\Users\junki\AppData\Local\Microsoft\Windows\INetCache\Content.Outlook\TJ0464QP\atsushi.kurotani@riken.jp)

^3^ Department of Information Systems, Niigata University of International and Information Studies, 3-1-1 Mizukino, Nishi-ku, Niigata-shi, Niigata 950-2292, Japan; [chikaya@nuis.ac.jp](file:///C:\Users\hkzig\AppData\Local\Packages\microsoft.windowscommunicationsapps_8wekyb3d8bbwe\LocalState\Files\S0\5\Attachments\chikaya@nuis.ac.jp)

^4^ Graduate School of Bioagricultural Sciences, Nagoya University, 1 Furo-cho, Chikusa-ku, Nagoya, Aichi 464-0810, Japan

***** Correspondence: [jun.kikuchi@riken.jp](mailto:jun.kikuchi@riken.jp); Tel.: +81-45-508-7220

**Supplementary material**

In this document, we provide the following information:

1. Details of time-frequency simulation method
2. Bayesian optimization
3. NMR measurements
4. Thermal analysis
5. Market basket analysis (MBA)
6. Self-organizing map (SOM)
7. References
8. Figures and tables

Analytical tools are available at <http://dmar.riken.jp/matrigica/>.

# **1.** Details of time-frequency simulation method

The equations for *T*_2_ relaxation used for the time domain fitting are given below (Equation (S1)).

$M\left( t \right)=M_{0}^{'}exp\left\{ -\left( \frac{t}{T_{2}} \right)^{W} \right\}$ (S1)

It was formulated with the parameters M_0_' (intensity), *t* (time), *T*_2_ (*T*_2_ transverse relaxation), and W (Weibull coefficient). The fitting was done so that W=1 for the Mobile component, W=2 for the Rigid component, and 1<W<2 for the Intermediate component.

It is known that FID can distinguish and quantify the signal contribution of each phase by the difference of time-averaged dipole-dipole coupling in rigid, intermediate and mobile domains^1^. Utilizing this mechanism, we performed a fitting based on *T*_2_ relaxation time information.

The following equation is used for the fitting of the frequency information of the four domain components (Equation (S2-5)).

【Mobile domain (Lorentzian function)】

$L\left( x \right)=M_{A}\left( \frac{\gamma}{\pi\left( x^{2}+\gamma^{2} \right)} \right)$ (S2)

【Intermediate (Mobile) domain】

$IM\left( x \right)=MAPE-L\left( x \right)$

$\sim\left[ \frac{A_{1}^{IM}}{\sigma^{IM}\sqrt{2\pi}} exp\left\{ -\frac{x^{2}}{{2\sigma^{IM}}^{2}} \right\}+\left( \frac{A_{2}^{IM}\gamma^{IM}}{\pi\left( x^{2}+{\gamma^{IM}}^{2} \right)} \right) \right]$ (S3)

【Rigid domain (Gaussian function)】

$G\left( x \right)=M_{B}\left[ \frac{1}{\sigma\sqrt{2\pi}} exp\left\{ -\frac{x^{2}}{{2\sigma}^{2}} \right\} \right]$ (S4)

【Intermediate (Rigid) domain】

$IR\left( x \right)=DQ-G(x)$

$\sim\left[ \frac{A_{1}^{IR}}{\sigma^{IR}\sqrt{2\pi}} exp\left\{ -\frac{x^{2}}{{2\sigma^{IR}}^{2}} \right\}+\left( \frac{A_{2}^{IR}\gamma^{IR}}{\pi\left( x^{2}+{\gamma^{IR}}^{2} \right)} \right) \right]$ (S5)

M_A_ and M_B_ are the spectral intensity, γ is the half-width at half-maximum in the Lorentzian function, σ is the half-width at half-maximum in the Gaussian function, and x is the frequency information. the mobile component is the Lorentzian function, and the rigid component is the Gaussian function. The intermediate component is calculated from the difference between MAPE and DQ-filter data.

In order to calculate the ratio between the mobile and intermediate (mobile) components obtained from the MAPE filtered spectra (MAPE), and between the rigid and intermediate (rigid) components obtained by the DQ filtered data (DQ), the calculation errors between the four domain components and the ^1^H-static spectra (Static) were calculated using the following equation (Equation (S6)).

$Calculation error= Anisotropy - \left( \alpha\times\left( M_{Mobile}+M_{IM} \right)+\beta\times\left( M_{IR}+M_{Rigid} \right) \right)$ (S6)

The purpose here is to eliminate the difference in the magnitude of the measurement intensity between the MAPE Filtered data and the DQ Filtered data, which removes the spectrum of a particular component of the static spectrum. This allows us to calculate the exact four domain components contained in the static spectrum.

After giving the α and β parameters that minimize the error, we created a 3D model of the four domain components based on the frequency and *T*_2_ relaxation time information.

The domain component ratios contained in the polymer material were calculated using the following equation (Equation (S7-10)).

$M_{mobile}=\frac{M\left( t \right)L\left( x \right)}{M_{0}^{'}}$ (S7)

$M_{intermediate\left( mobile \right)}=\frac{M\left( t \right)IM\left( x \right)}{M_{0}^{'}}$ (S8)

$M_{intermediate\left( rigid \right)}=\frac{M\left( t \right)IR\left( x \right)}{M_{0}^{'}}$ (S9)

$M_{rigid}=\frac{M\left( t \right)G\left( x \right)}{M_{0}^{'}}$ (S10)

A detailed description of the time-frequency simulation method is given in Figure S1. The FIDs obtained from the MAPE and DQ filters, as well as the Fig. for calculation error minimization shown in equation (6), are also shown in Figure S1.

The weighted average (WA) of the $T_{2}$ relaxation times for a single polymer material was calculated using the following equation (Equation (11)).

$$WA=\{M_{Mobile}T_{2}\left( Mobile \right)+ M_{IM}T_{2}^{IM}+ M_{IR}T_{2}^{IR}$$

$+M_{Rigid}T_{2}\left( Rigid \right)\}/(M_{mobile}+M_{IM} + M_{IR}+M_{Rigid})$ (S11)

# **2. Bayesian optimization for automated spectral simulation**

Bayesian optimization was utilized for searching the ratio of mobile and rigid component with the equation 6 in main text to reduce the error between the fitted NMR spectral simulation values and the static data^2^. The acquisition function was updated by Gaussian process. The search range of ratio is from 0 as the minimum value to 10 as the maximum value for each exploratory variable, which value is floating point number. Before, Bayesian optimization of the fitting of Gaussian process, 5 points were randomly selected as the initial variables to sample the target function. The iteration count of Bayesian optimization was used 25.

# **3. NMR measurement**

The solid-state NMR (ssNMR) data were acquired using an Avance III HD-500 spectrometer (Bruker Corp., Billerica, MA, USA) equipped with a double-resonance 4.0 mm MAS probe. The polymer materials were measured using ^1^H-static, MAPE-filtered and DQ-filtered ssNMR.

# **4. Thermal analysis**

Thermogravimetry (TG) and differential thermal analysis (DTA) measurements were conducted using an EXSTAR TG/DTA 6300 (SII NanoTechnology Inc., Tokyo, Japan) instrument. Approximately 10 mg of samples was individually vaporized at 5 °C/min from 40 to 500 °C in a nitrogen atmosphere. The *T*_m_ and *T*_d_ were determined as the endothermic peak in DTA curves and the peak of weight loss in Derivative Thermogravimetry (DTG) curves. Differential scanning calorimetry (DSC) was conducted using a DSC3500A (NETZSCH Geratebau GmbH, Selb, Germany). Approximately 1.5 mg of samples was individually measured at the following steps at 10 °C/min from 25 to −30 °C, at 10 °C/min from −30 to 200 °C, and at 20 °C/min from 200 to 25 °C in a nitrogen atmosphere. The *T*_g_ was determined as an endothermic peak during heating.

# **5. Market basket analysis (MBA)**

Association rules were determined using criterion values of support, confidence, and lift. An association rule is thus expressed in the form X ⇒ Y, where X ∩ Y = ϕ. Let X be a set of some variables in Item (I), I = {i1, i2, ···, im} be a set of all possible variables, likewise Y be a set of some variables in I. Support is the probability of X and Y co-occurring in the transaction data set.

The confidence of the rule X ⇒ Y is the conditional probability of observing Y given that X is present in a transaction. The lift of the rule X ⇒ Y is the ratio of the support if X and Y are independent. Therefore, higher lift values imply a high probability of event Y in the case of condition X. Since lift values <1 do not correlate (independent relationship) between X and Y as association rules. The market basket analysis was performed on R with the package “arules”^3-5^ (<https://cran.r-project.org/web/packages/arules/index.html>). The market basket analysis parameters were set as “maxlen (maximum size of mined frequent item sets) = 2” and “lift > 1”. The association network was depicted via the use of the Cytoscape program ([https://www.cytoscape.org/)](https://www.cytoscape.org/)6)^[6](https://www.cytoscape.org/)6)^.

# **6. Self-organizing map (SOM)**

Self-organizing maps (SOMs) of Kohonen, also known as a Kohonen map^7^, is a neural-network model for exploring and visualizing multi-dimensional patterns in high-dimensional data sets. SOMs can be considered as a non-linear mapping technique, which identifies clusters in an unsupervised way within data sets without the rigid assumptions of linearity or normality associated with traditional statistical techniques. Unlike other neural networks, the SOM approach does not require any target output and it is an unsupervised pattern recognition method such as clustering and PCA^8^. In this study, all calculations concerning SOM were performed by the Kohonen library on the R platform.

# **7. References**

1. Schafer, M.; Yuan, S. C.; Petzold, A.; Perez-Camargo, R. A.; Muller, A. J.; Thurn-Albrecht, T.; Saalwachter, K.; Schmidt-Rohr, K. *Macromolecules* **2021**, *54*, 835-845.

2. Yamawaki, R.; Tei, A.; Ito, K.; Kikuchi, J. *Appl. Sci.* **2021**, 11 2820.

3. Shiokawa, Y., Misawa, T., Date, Y. and Kikuchi, J. *Anal. Chem.* **2016**, 88, 2714-2719.
4. Ito, K.; Sakata, K.; Date, Y.; Kikuchi, J. *Anal. Chem.* **2014**, *86*, 1098-1105.
5. Michael, H.; Sundheer, C.; Kurt, H.; Christian, B. *J. Mach. Learn. Res.* **2011**, 12, 2021−2025.
6. Shannon, P.; Markiel, A.; Ozier, O.; Baliga, N. S.; Wang, J. T.; Ramage, D.; Amin, N.; Schwikowski, B.; Ideker, T. *Genome Res.* **2003**, 13, 2498−2504

7. Kohonen, T. **1989** Self-organization and associative memory: 3rd edition, Springer-Ve rlag New York, Inc.
8. Voyslavov, T., Tsakovski, S., and Simeonov, V. **2012** Surface water quality assessmen t using self-organizing maps and Hasse diagram technique, Chemometrics and Intelligen t Laboratory Systems 118, 280-286.

# **8. Figures and tables**

**
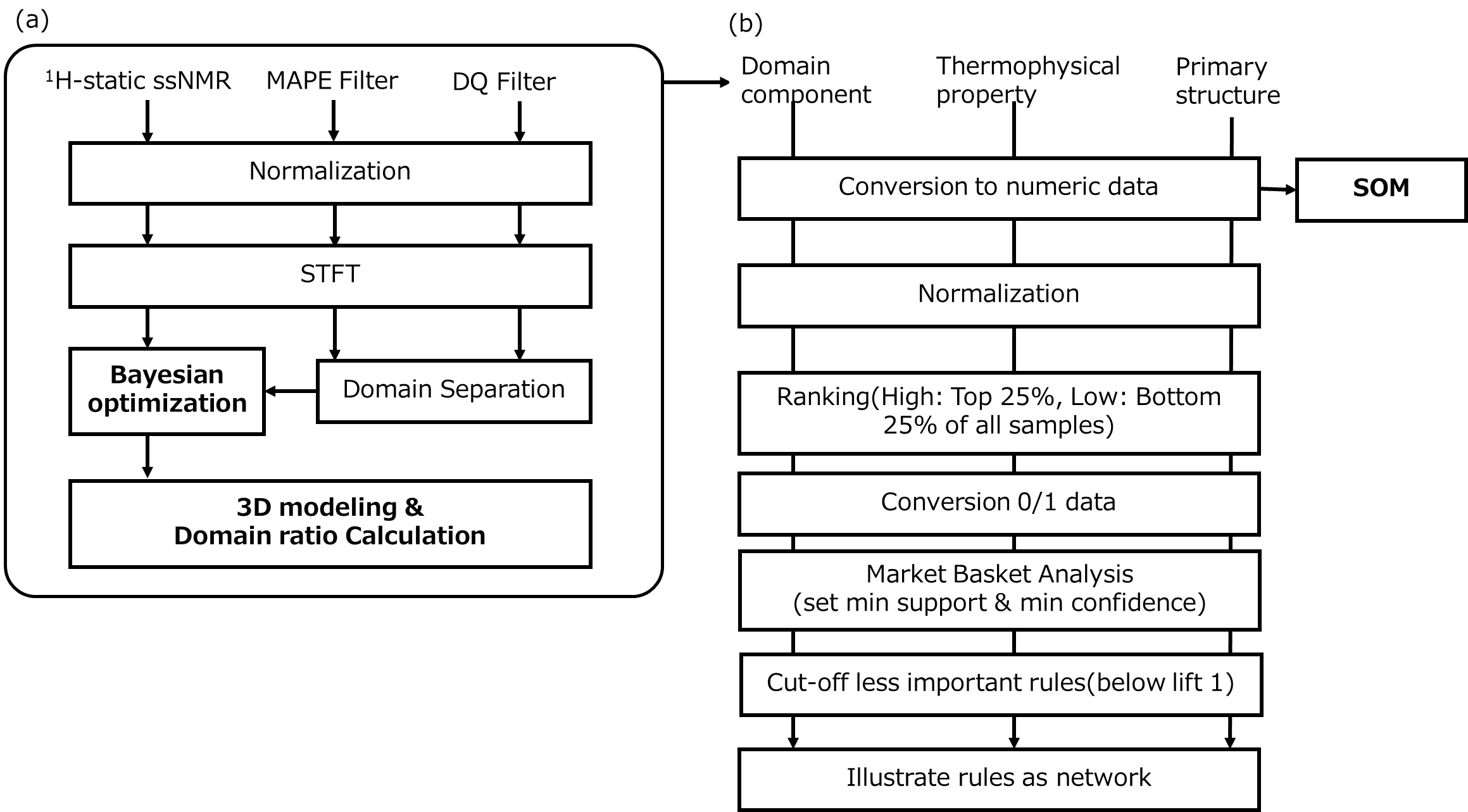
**

## **Figure S1.** Analytical flow of materials informatics approach using domain modelling for exploring structure-property relationships of polymers.

(a) The input spectrum is subjected to normalization, short-time Fourier transform, domain component separation, and Bayesian optimization. In this process, the domain ratios are calculated from the ^1^H-stationary solid-state NMR (ssNMR) spectra using the domain information from the MAPE-filtered and DQ-filtered spectra. (b) To exploring for structure-property relationships, we perform integrated analysis of domain ratios and thermophysical properties and primary structure information. After converting the integrated data into numerical values, SOM is performed. After the integrated data is converted to numerical values, normalized, ranked, and converted to 0/1 data, MBA is performed. The relationships obtained from the MBA are narrowed down to important factors, and a network diagram showing the relationships is created.


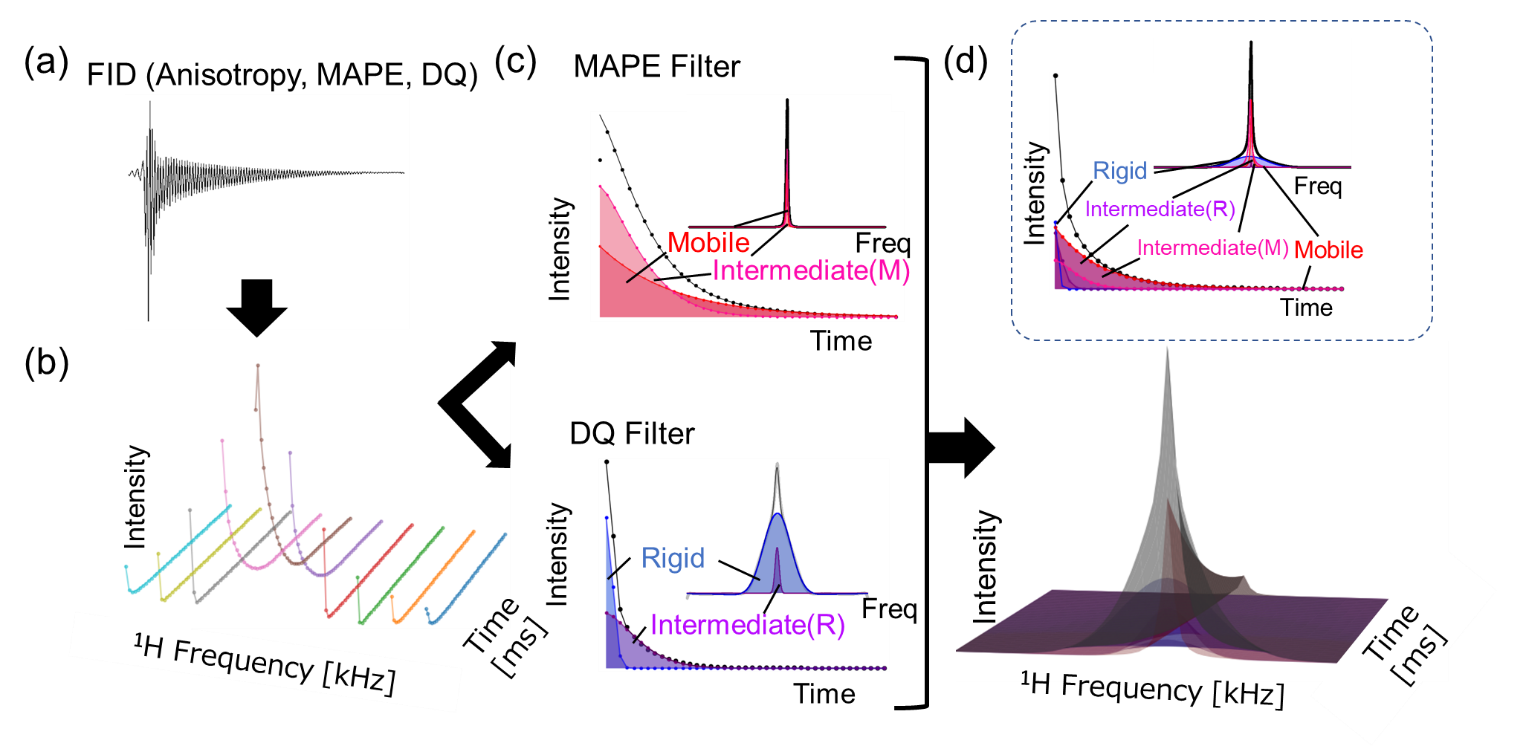


## **Figure S2.** Analytical flow of time-frequency simulation method for a set of representative data.

(a) An example of FID data. (b) Short-time Fourier transform for NMR spectrogram. (c) Fitting results for frequency and relaxation time information obtained by MAPE filter (red region of Mobile, pink region of Intermediate (Mobile; M), black plots of real data) and DQ filter (purple region of Intermediate (Rigid; R), blue region of Rigid black plots of real data). (d) 3D domain component simulation model based on the fitting results of those four components.


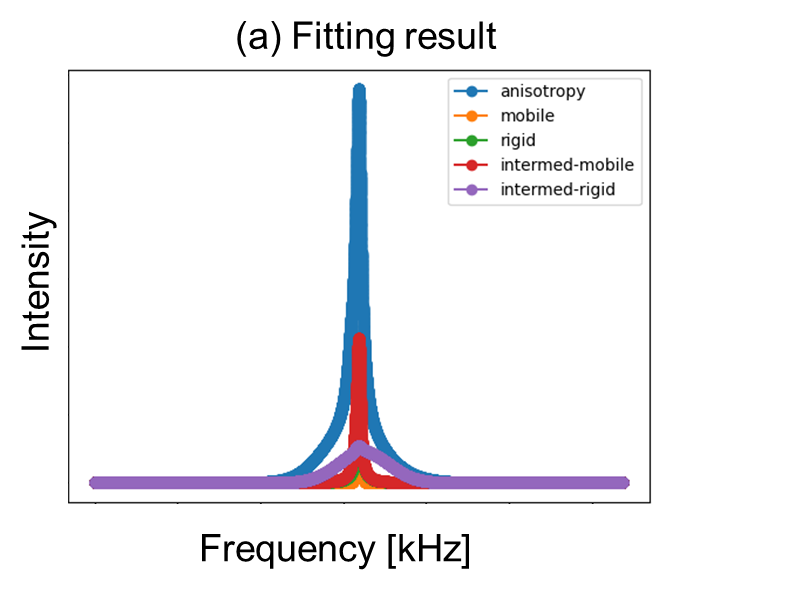


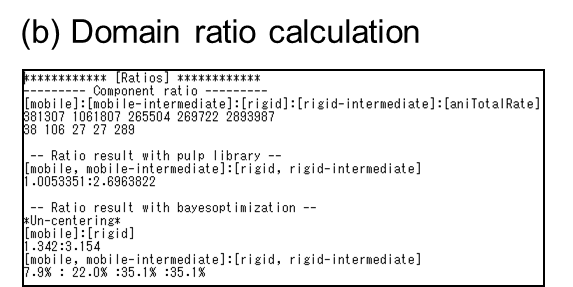


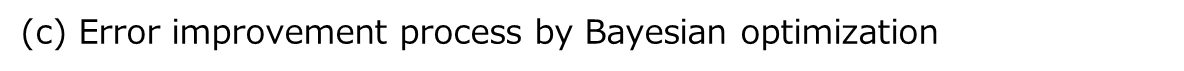


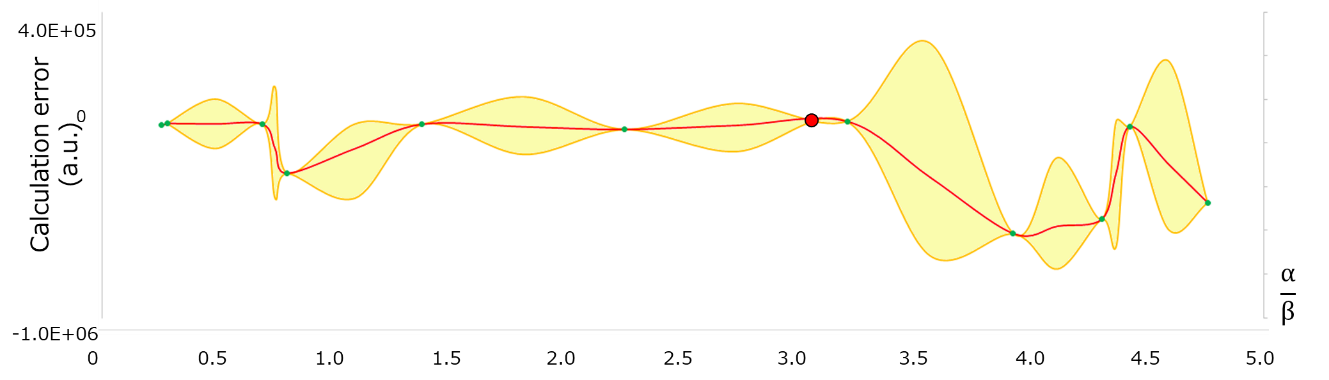


## **Figure** **S3.** 3D simulation model automation tool based on domain component separated parameters by fitting.

By inputting the DQ filtered data, MAPE filtered data, and static data measured by NMR and the output destination. (a) Frequency fitting results using the tool. (b) Component ratio calculation using the tool. (c) Error improvement process by Bayesian optimization. α indicates the ratio of Mobile to Intermediate (Mobile), and β indicates the ratio of Rigid to Intermediate (Rigid). The tool was created using Python.

**
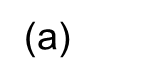
**

**
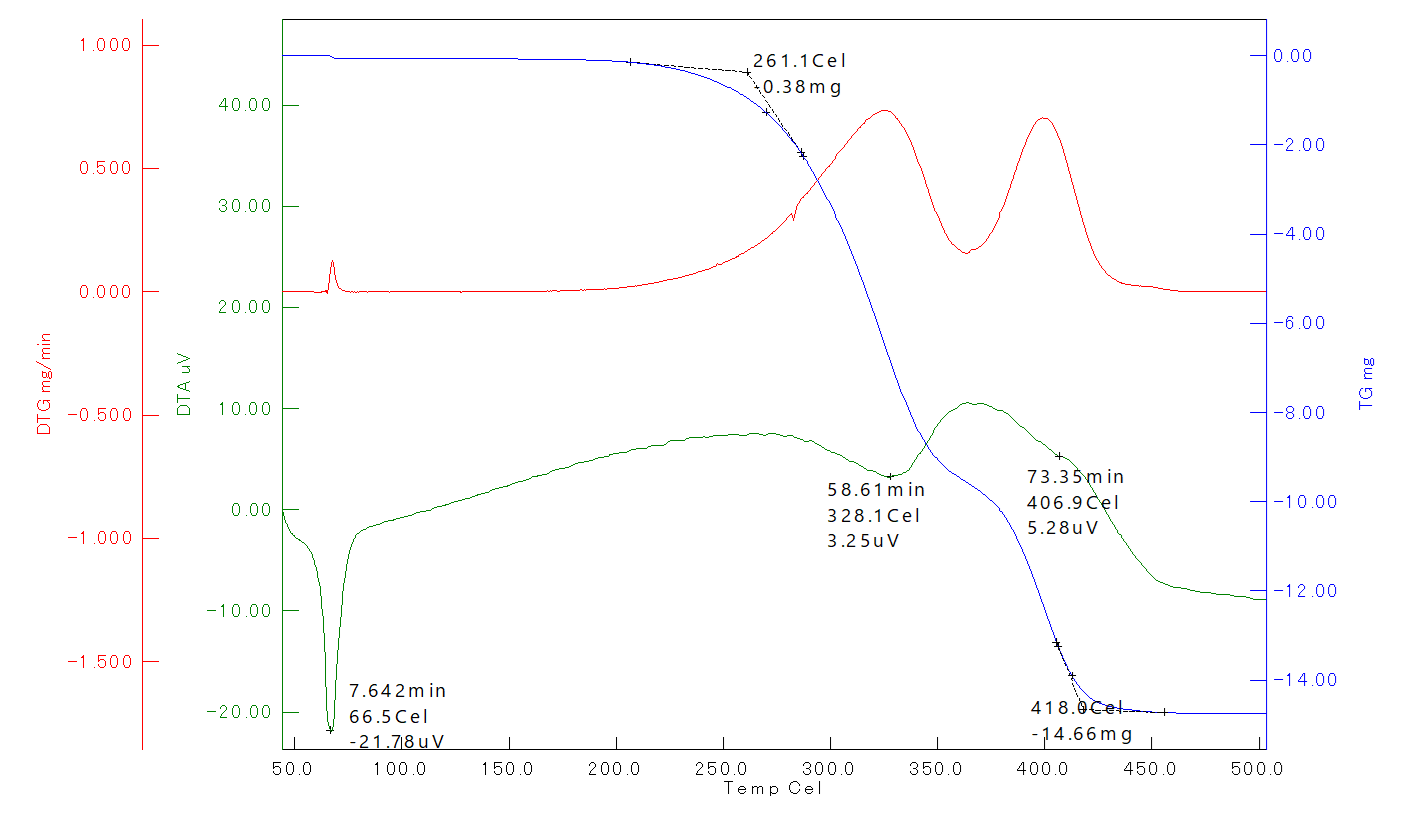
**


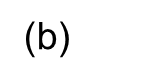


**
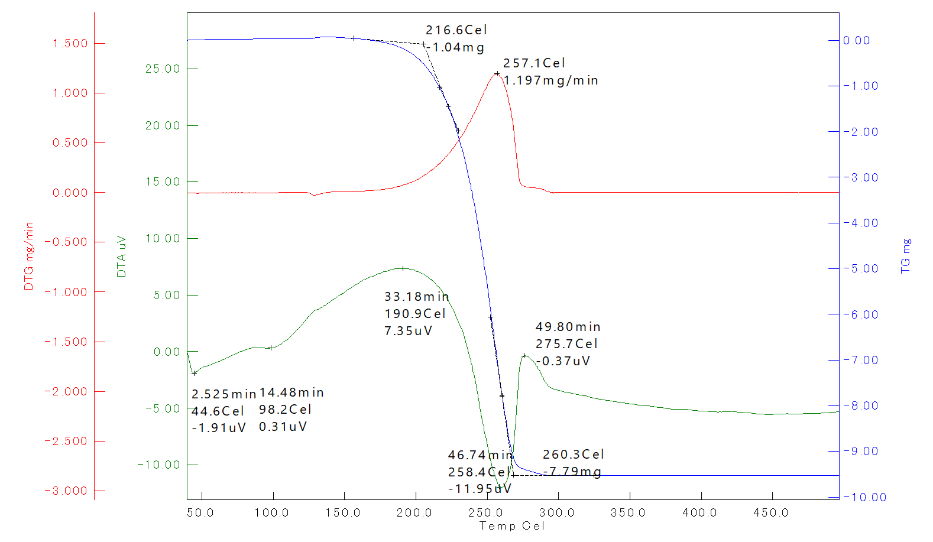
**

## **Figure S4.** Typical examples of TG/DTA/DTG Parameters.

Thermal analysis data of (a) PCL (Sample ID 15) and (b) PLA (Sample ID 60) are shown as typical polymer materials. The red line is the DTG curve, the blue line is the TG curve, and the green line is the DTA curve. In each curve, numerical data of parameters related to polymer-specific properties are entered.


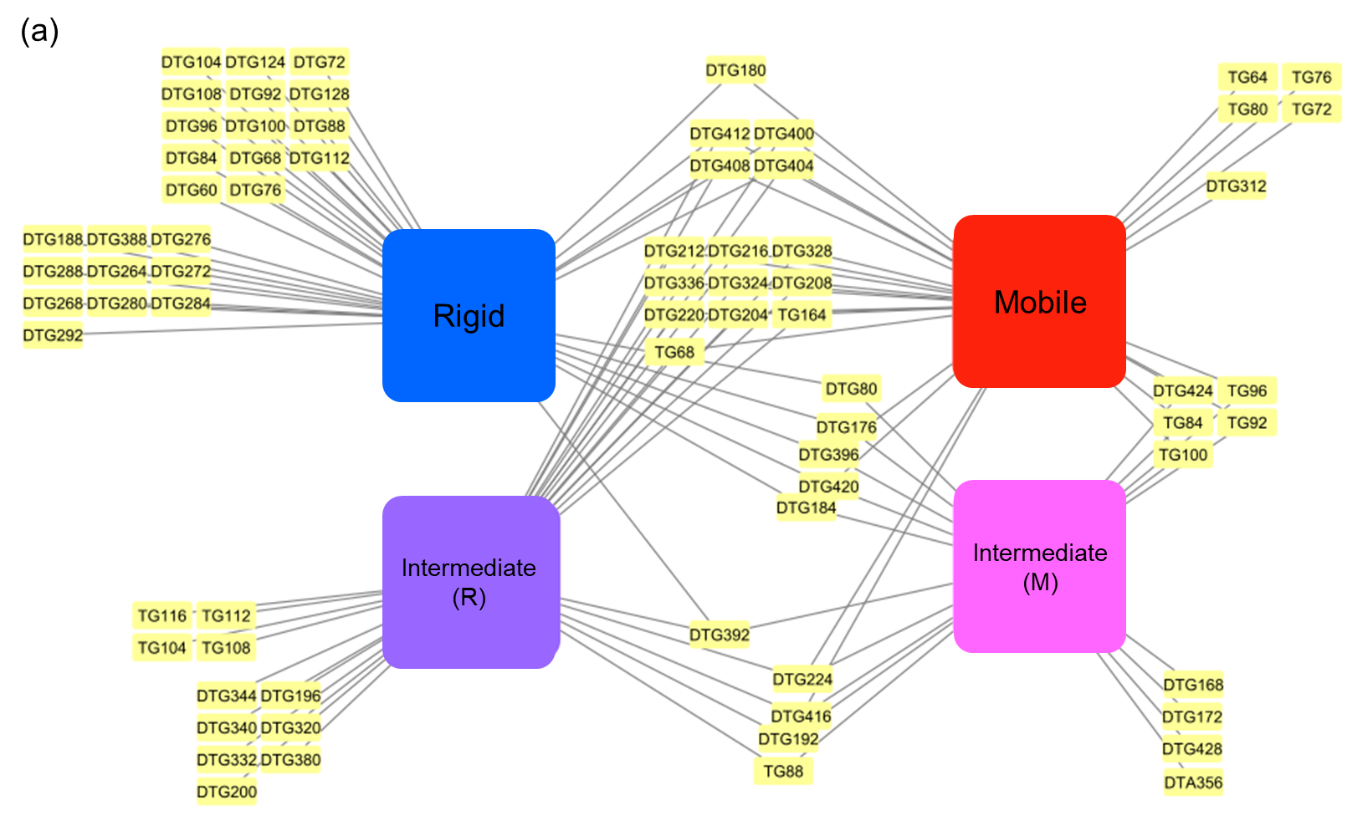


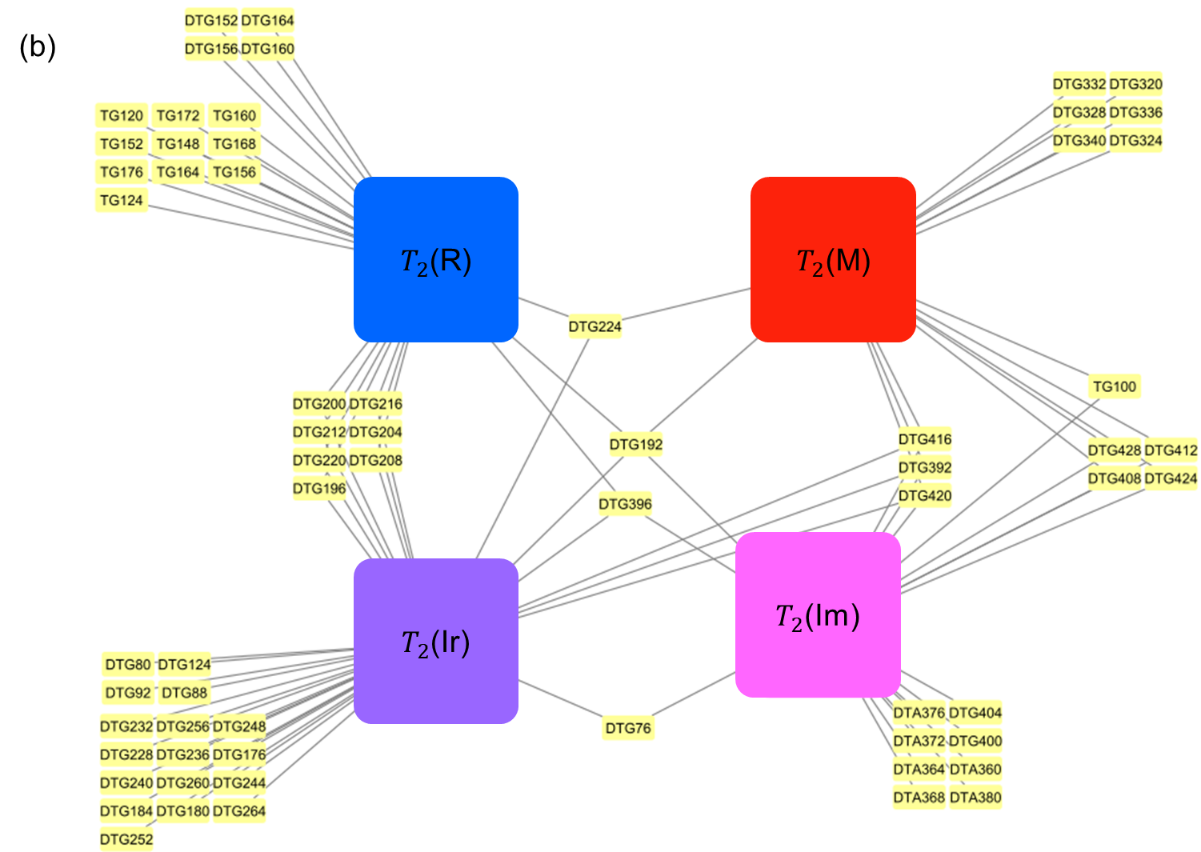


## **Figure S5****.** MBA using physical property data and domain composition information.

The results of market basket analyses using thermal analysis data, NMR spectral data, and domain component information. (a) 3D model volume values. Blue: Rigid, Purple: Intermediate(Rigid), Pink: Intermediate(Mobile), Red: Mobile, Yellow: DTG, DTA, TG are parameters at each temperature. (b) *T*_2_ relaxation time information are presented. Blue: *T*_2_(R) (*T*_2_ of rigid component), Purple: *T*_2_(Ir) (*T*_2_ of intermediate(rigid) component), Pink: *T*_2_(Im) (*T*_2_ of intermediate(mobile) component), Red: *T*_2_(M) (*T*_2_ of mobile component), Yellow: DTG, DTA, TG are parameters at each temperature.

## **Table S1****.** Measurement method and analytical method of polymer materials.

This table indicates material sample ID, abbreviation of polymer name, manufacturer's name, measurement method (TG-DTA, DSC, DMA, IR, CP-MAS), pretreatment method of polymer materials, each sample and experimental item (CPMG, solid-echo, SOM, MBA, Fitting).

m1: Cutting with scissors, m2: Freeze grinding, m3: Cutting with cutter, m4: Freeze cutter, m5: Remove adhesive surface, m6: Crush with mortar, m7: native samples, circle: analyzed, bar: no analyzed, PLA: poly(lactic acid), PGA: poly(glycolic acid), PE: polyethene, PP: polyethene, PES: polyethersulfone, PVDF: poly(vinylidene fluoride), PHBH: poly(3-hydroxybutyrate-co-3-hydroxyhexanoate), PBS: poly[(butane-1,4-diol)-alt-(succinic acid)], PBSA: poly[(butane-1,4-diol)-alt-(adipic acid)], PVA: poly(vinyl alcohol), PS: polystyrene, PET: poly(ethylene terephthalate), PVDC: poly(vinylidene chloride), PI: polyimide, PEN: poly(ethylene naphthalene-2,6-dicarboxylate), PBAT: poly{[(butane-1,4-diol)-alt-(adipic acid)]-co-[(butane-1,4-diol)-alt-(terephthalic acid)]}, PBAT: poly[(butane-1,4-diol)-alt-(terephthalic acid)]: PBS(A)/T, PVC: poly(vinyl chloride), ABS: poly(acrylonitrile-co-butadiene-co-styrene), PBS: poly[(butane-1,4-diol)-alt-(succinic acid)], SBC: poly(styrene-co-butadiene), PC: 2,2'-dimethylbisphenol A polycarbonate, PETG: poly[(ethylene glycol;cyclohexane-1,3-dimethanol)-alt-(terephthalic acid)], TPU: Thermoplastic Polyurethane, CoPA: poly(6-aminocaproic acid-co-hexamethylene adipamide), Nylon: poly(hexano-6-lactam), SBR: poly(styrene-co-butadiene), NR: cis-1,4-polyisoprene, PES: Poly(ethylene succinate), Y / N is with or without measurement data or unknown. In the SOM column, the order of the samples used in the SOM is listed.

| **Sample ID** | **Polymer name** | **manufacturer's name** | **TG-DTA** | **DSC** | **DMA** | **IR** | **CP-MAS** | **Pretreatment** | **CPMG** | | **solid-echo** | **SOM** | **MBA** | **Fitting** |  |
| --- | --- | --- | --- | --- | --- | --- | --- | --- | --- | --- | --- | --- | --- | --- | --- |
| S1-1 | PET | N | Y | Y | N | Y | Y | m1 | Y | | Y | 1 | Y | N |  |
| S1-2 | PET | CocaNColaNBottlersNJapanNInc. | Y | Y | N | Y | Y | m1 | Y | | Y | 2 | Y | N |  |
| S1-3 | PET | YUTAKANSHOJINCO.,NLTD. | Y | N | N | Y | N | m1 | Y | | Y | N | N | N |  |
| S1-4 | PET | YUTAKANSHOJINCO.,NLTD. | Y | Y | N | Y | Y | m1 | Y | | Y | N | N | N |  |
| S1-5 | PET | TorayNIndustries,NInc | Y | Y | N | Y | Y | m1 | Y | | Y | 3 | Y | N |  |
| S1-6 | PET | MarusanNHashimotoNCo,NLtd | Y | Y | N | Y | Y | m1 | Y | | Y | 4 | Y | N |  |
| S1-7 | PET | MatsumuraNKogeiNCo.,NLtd | Y | Y | N | Y | Y | m1 | Y | | Y | N | Y | N |  |
| S1-8 | PE | AS ONE CORPORATION | Y | Y | N | Y | Y | m1 | Y | | Y | 5 | Y | N |  |
| S1-9 | PE | RINREI TAPE Co.,LTD | Y | N | N | Y | Y | m1→m5 | Y | | Y | 6 | Y | N |  |
| S1-10 | PE | SEISANNIPPONSHANLTD | Y | Y | N | Y | Y | m1 | Y | | Y | 7 | Y | N |  |
| S1-11 | PE | SeihoNCo.,NLtd | Y | Y | N | Y | Y | m1 | Y | Y | | 9 | Y | N | |
| S1-12 | PE | FukusukeNkogyoNCo.,NLtd | Y | Y | N | Y | Y | m1 | Y | | Y | 10 | Y | N |  |
| S1-13 | PE | N | Y | Y | N | Y | Y | m1 | Y | | Y | 11 | Y | N |  |
| S1-14 | PHBH | Yamazen corporation | Y | Y | N | Y | Y | m3 | Y | | Y | 59 | Y | Y |  |
| S1-15 | PHBH | kaneka | Y | N | N | Y | N | m7 | N | | N | N | Y | Y |  |
| S1-16 | PHBH | kaneka | Y | N | N | Y | Y | m7 | Y | | Y | N | Y | Y |  |
| S1-17 | PHBH | kaneka | Y | N | N | Y | Y | m7 | Y | | Y | N | Y | Y |  |
| S1-18 | PHBH | kaneka | Y | N | N | Y | Y | m7 | Y | | Y | N | Y | Y |  |
| S1-19 | PHBH | kaneka | Y | N | N | Y | Y | m7 | Y | | Y | N | Y | Y |  |
| S1-20 | PHBH | kaneka | Y | N | N | Y | Y | m7 | Y | | Y | N | Y | Y |  |
| S1-21 | PHBH | kaneka | Y | N | N | Y | Y | m7 | Y | | Y | N | Y | Y |  |
| S1-22 | PHBH | kaneka | Y | N | N | Y | Y | m7 | Y | | Y | N | Y | Y |  |
| S1-23 | PES | Merck | Y | Y | N | Y | Y | m1 | Y | | Y | N | Y | N |  |
| S1-24 | PES | Aldrich | Y | N | N | Y | N | m7 | N | | N | 61 | Y | Y |  |
| S1-25 | PES | TCI | Y | N | N | Y | N | m7 | N | | N | 62 | N | N |  |
| S1-26 | PBS | ShinNEtsu Polymer Co., Ltd. | Y | Y | N | Y | Y | m2 | Y | | Y | 36 | Y | N |  |
| S1-27 | PBS | Nature3D | Y | N | N | Y | Y | m2 | Y | | Y | 38 | Y | Y |  |
| S1-28 | PBS | BASF | Y | N | N | Y | Y | m7 | Y | | Y | N | Y | N |  |
| S1-29 | PBAT | UNICNCO. | Y | Y | N | Y | Y | m1 | Y | | Y | 12 | Y | N |  |
| S1-30 | PBAT | AchillesNCo.,NLtd | Y | Y | N | Y | Y | m1 | Y | | Y | 13 | Y | N |  |
| S1-31 | PBAT | BASF | Y | N | N | Y | N | m7 | N | | N | N | Y | Y |  |
| S1-32 | PBAT | BASF | Y | N | N | Y | Y | m7 | Y | | Y | N | Y | Y |  |
| S1-33 | PBSA | kiracs Co., Ltd | Y | Y | N | Y | Y | m1 | Y | | Y | 39 | Y | N |  |
| S1-34 | PBSA | ChukohNChemicalNIndustries,NLtd. | Y | Y | N | Y | Y | m1 | Y | | Y | 42 | Y | N |  |
| S1-35 | PBSA | kiracsNCo.,NLtd | Y | Y | N | Y | Y | m1 | Y | | Y | 40 | Y | N |  |
| S1-36 | PBSA | TsujinoNPlasticsNIndustryNCo. | Y | Y | N | Y | Y | m1 | Y | | Y | 41 | Y | N |  |
| S1-37 | PBSA | N | Y | N | N | Y | N | m7 | N | | N | N | N | Y |  |
| S1-38 | PBSA | N | Y | N | N | Y | Y | m1 | Y | | Y | N | Y | Y |  |
| S1-39 | PBSA | N | Y | N | N | Y | Y | m1 | Y | | Y | N | Y | Y |  |
| S1-40 | PCL | Infin8NInc | Y | Y | N | Y | Y | m4 | Y | | Y | 43 | Y | Y |  |
| S1-41 | PCL | KENISNCo.,NLtd | Y | Y | N | Y | Y | m1 | Y | | Y | 44 | Y | N |  |
| S1-42 | PCL | FUJIFILMNWakoNPureNChemicalNCorporation | Y | Y | N | Y | Y | m2 | Y | | Y | 45 | Y | N |  |
| S1-43 | PCL | XYZprinting | Y | N | N | Y | Y | m2 | Y | | Y | 46 | N | Y |  |
| S1-44 | PCL | XYZprinting | Y | N | N | Y | Y | m1 | Y | | Y | 47 | Y | N |  |
| S1-45 | PCL | polysciences | Y | N | N | Y | Y | m7 | Y | | Y | 48 | Y | Y |  |
| S1-46 | PCL | polysciences | Y | N | N | Y | Y | m7 | Y | | Y | 49 | Y | Y |  |
| S1-47 | PCL | polysciences | Y | N | N | Y | Y | m7 | Y | | Y | 50 | Y | Y |  |
| S1-48 | PCL | polysciences | Y | N | N | Y | Y | m7 | Y | | Y | N | Y | Y |  |
| S1-49 | PCL | polysciences | Y | N | N | Y | Y | m1 | Y | | Y | N | Y | Y |  |
| S1-50 | PCL | polysciences | Y | N | N | Y | Y | m1 | Y | | Y | N | Y | Y |  |
| S1-51 | PCL | polysciences | Y | N | N | Y | Y | m1 | Y | | Y | N | Y | Y |  |
| S1-52 | PCL | polysciences | Y | N | N | Y | Y | m1 | Y | | Y | N | Y | Y |  |
| S1-53 | PCL | polysciences | Y | N | N | Y | Y | m1 | Y | | Y | N | Y | Y |  |
| S1-54 | PLA | Office Media | Y | Y | N | Y | Y | m1 | Y | | Y | 14 | Y | N |  |
| S1-55 | PLA | Ajinomoto Co., Inc. | Y | Y | N | Y | Y | m1 | Y | | Y | 15 | Y | N |  |
| S1-56 | PLA | Ajinomoto Co., Inc. | Y | N | N | Y | Y | m1 | Y | | Y | 16 | Y | N |  |
| S1-57 | PLA | kunimune | Y | N | N | Y | Y | m1→m5 | Y | | Y | 17 | Y | N |  |
| S1-58 | PLA | kunimune | Y | Y | N | Y | Y | m6 | Y | | Y | 18 | Y | N |  |
| S1-59 | PLA | RikenNfabroNCorporation. | Y | Y | N | Y | Y | m1 | Y | | Y | 19 | Y | N |  |
| S1-60 | PLA | belart | Y | Y | N | Y | Y | m3 | Y | | Y | 20 | Y | N |  |
| S1-61 | PLA | entec | Y | Y | N | Y | Y | m3 | Y | | Y | 21 | Y | N |  |
| S1-62 | PLA | LION | Y | Y | N | Y | Y | m1 | Y | | Y | N | Y | N |  |
| S1-63 | PLA | MUTOHNINDUSTRIESNLTD. | Y | Y | N | Y | Y | m1 | Y | | Y | 22 | Y | N |  |
| S1-64 | PLA | miyacoNLTD | Y | Y | N | Y | Y | m1 | Y | | Y | 23 | Y | N |  |
| S1-65 | PLA | FujiNchemicalNltd | Y | Y | N | Y | Y | m1 | Y | | Y | 24 | Y | N |  |
| S1-66 | PLA | DARCOS | Y | Y | N | Y | Y | m1 | Y | | Y | 25 | Y | N |  |
| S1-67 | PLA | Arromic | Y | Y | N | Y | Y | m2 | Y | | Y | 26 | Y | N |  |
| S1-68 | PLA | EXCEL | Y | Y | N | Y | Y | m4 | Y | | Y | 64 | Y | N |  |
| S1-69 | PLA | klarkNCo, | Y | Y | N | Y | Y | m1 | Y | | Y | 27 | Y | N |  |
| S1-70 | PLA | goodmediaNCo, | Y | Y | N | Y | Y | m4 | Y | | Y | 65 | Y | N |  |
| S1-71 | PLA | AsahiNKaseiNpacksNco.,Nltd | Y | Y | N | Y | Y | m1 | Y | | Y | 28 | Y | N |  |
| S1-72 | PLA | UNITIKA | Y | Y | N | Y | Y | m1 | Y | | Y | 29 | Y | N |  |
| S1-73 | PLA | UNITIKA | Y | Y | N | Y | Y | m1 | Y | | Y | 30 | Y | N |  |
| S1-74 | PLA | zenmiNCo.,NLtd | Y | Y | N | Y | Y | m1 | Y | | Y | N | Y | N |  |
| S1-75 | PLA | 18402N10 | Y | Y | N | Y | Y | m2 | Y | | Y | N | Y | N |  |
| S1-76 | PLA | noztek | Y | N | N | Y | Y | m3 | Y | | Y | N | Y | N |  |
| S1-77 | PLA | Nature3D | Y | N | N | Y | Y | m2 | Y | | Y | N | Y | N |  |
| S1-78 | PLA | Polymaker | Y | N | N | Y | Y | m1 | Y | | Y | 31 | Y | Y |  |
| S1-79 | PLA | polysciences | Y | N | N | Y | Y | m2 | Y | | Y | 32 | Y | N |  |
| S1-80 | PLA | Nature3D | Y | N | N | Y | N |  | N | | N | N | N | N |  |
| S1-81 | Nylon | taulman3D | Y | N | N | Y | Y | m2 | Y | | Y | 33 | Y | N |  |
| S1-82 | Nylon | TokocmonNLimitedNCompany | Y | N | N | Y | Y | m1 | Y | | Y | 34 | Y | N |  |
| S1-83 | Nylon | SemitecNCo.,NLtd | Y | N | N | Y | Y | m1 | Y | | Y | 35 | Y | N |  |
| S1-84 | PEN | DuPontNTeijinNAdvancedNPapersNCo.NLTD. | Y | Y | N | Y | Y | m1 | Y | | Y | 63 | Y | N |  |
| S1-85 | PGA | GUNZE LIMITED | Y | Y | N | Y | Y | m1 | Y | | Y | 60 | Y | N |  |
| S1-86 | PP | N | Y | Y | N | Y | Y | m3→m2 | Y | | Y | N | Y | N |  |
| S1-87 | PVDF | Merck | Y | Y | Y | Y | Y | m1 | Y | | Y | 57 | Y | N |  |
| S1-88 | PVA | Green Letter Contracts | Y | Y | N | Y | Y | m1 | Y | | Y | 53 | Y | N |  |
| S1-89 | PS | Ajinomoto Co., Inc. | Y | Y | N | Y | Y | m1 | Y | | Y | 58 | Y | N |  |
| S1-90 | PP | N | Y | Y | N | Y | Y | m1 | Y | | Y | N | Y | N |  |
| S1-91 | PS | N | Y | Y | N | Y | Y | m1 | Y | | Y | N | Y | N |  |
| S1-92 | PP | YUTAKANSHOJINCO.,NLTD. | Y | N | N | Y | N | m1 | Y | | Y | 51 | N | N |  |
| S1-93 | PP | YUTAKANSHOJINCO.,NLTD. | Y | Y | N | Y | N | m1 | Y | | Y | 52 | N | N |  |
| S1-94 | PP | YUTAKANSHOJINCO.,NLTD. | Y | N | N | Y | Y | m1 | Y | | Y | N | N | N |  |
| S1-95 | PP | YUTAKANSHOJINCO.,NLTD. | Y | N | N | Y | N | m1 | Y | | Y | N | N | N |  |
| S1-96 | PP | YUTAKANSHOJINCO.,NLTD. | Y | Y | N | Y | N | m1 | Y | | Y | N | N | N |  |
| S1-97 | PP | YUTAKANSHOJINCO.,NLTD. | Y | N | N | Y | Y | m1 | Y | | Y | N | N | N |  |
| S1-98 | PP | YUTAKANSHOJINCO.,NLTD. | Y | N | N | Y | Y | m1 | N | | N | N | N | N |  |
| S1-99 | PP | YUTAKANSHOJINCO.,NLTD. | Y | N | N | Y | N | m1 | Y | | Y | N | N | N |  |
| S1-100 | PVDC | AsahiNKasei | Y | Y | N | Y | Y | m1 | Y | | Y | N | Y | N |  |
| S1-101 | PI | DUNPONTNTORAYNCO.,NLTD. | Y | N | N | Y | Y | m1 | Y | | Y | N | Y | N |  |
| S1-102 | PP,PE | Ajinomoto Co.,NInc. | Y | N | N | Y | Y | m2 | Y | | Y | N | Y | N |  |
| S1-103 | PP | Ajinomoto Co.,NInc. | Y | Y | N | Y | Y | m2 | Y | | Y | N | Y | N |  |
| S1-104 | BSNLA_copolymer | IwataniNMaterialsNCorporation | Y | Y | N | Y | Y | m1 | Y | | Y | N | Y | N |  |
| S1-105 | PBS(A)/T | MitsubishiNChemicalNAgriNDreamNCo.N,NLtd. | Y | Y | N | Y | Y | m1 | Y | | Y | N | Y | N |  |
| S1-106 | PE+10%BioPE | N | Y | Y | N | Y | Y | m1 | Y | | Y | 8 | Y | N |  |
| S1-107 | PA | AoiNCo.,Ltd | Y | Y | N | Y | Y | m1 | Y | | Y | 55 | Y | N |  |
| S1-108 | PVC | Okamoto | Y | Y | N | Y | Y | m1 | Y | | Y | N | Y | N |  |
| S1-109 | PVA | FUJIFILMNWakoNPureNChemicalNCorporation | Y | Y | N | Y | Y | m7 | Y | | Y | 54 | Y | N |  |
| S1-110 | ABS | noztek | Y | N | N | Y | Y | m2 | Y | | Y | N | Y | N |  |
| S1-111 | SBC | Nature3D | Y | N | N | Y | Y | m2 | Y | | Y | N | N | Y |  |
| S1-112 | PC | Polymaker | Y | N | N | Y | Y | m1 | Y | | Y | N | N | N |  |
| S1-113 | PC | Polymaker | Y | N | N | Y | Y | m1 | Y | | Y | N | N | N |  |
| S1-114 | PETG | Polymaker | Y | N | N | Y | Y | m1 | Y | | Y | N | N | N |  |
| S1-115 | TPU | Polymaker | Y | N | N | Y | Y | m1 | Y | | Y | 56 | N | N |  |
| S1-116 | CoPA | Polymaker | Y | N | N | Y | Y | m1 | Y | | Y | N | N | N |  |
| S1-117 | NR | ASNONENCORPORATION | Y | N | N | Y | Y | m1 | Y | | Y | N | Y | N |  |
| S1-118 | SBR | ASNONENCORPORATION | Y | N | N | Y | Y | m1 | Y | | Y | N | N | N |  |
| S1-119 | SBR | MakiguchiNrubberNCo.,NLtd | Y | N | N | Y | Y | m2 | Y | | Y | N | N | N |  |
| S1-120 | NR | KyowaNCo.,NLtd | Y | N | N | Y | Y | m2 | Y | | Y | N | N | N |  |

## **Table S2****.** List of domain component information.

The domain component information calculated by the component separation method is indicated as M: Mobile, I(M): Intermediate(Mobile), I(R): Intermediate(Rigid), R: Rigid, *T*_2_ (M), *T*_2_ (Im), *T*_2_ (Ir), *T*_2_ (R), and WA: Weighted average.

| Polymer name | Sample ID | M | I(M) | I(R) | R | *T*_2_ (M)  [s] | *T*_2_ (Im)  [s] | *T*_2_ (Ir)  [s] | *T*_2_ (R)  [s] | WA |
| --- | --- | --- | --- | --- | --- | --- | --- | --- | --- | --- |
| PHBH-1 | S2-1 | 909.45 | 414.12 | 5648.77 | 7830.75 | 0.000742 | 0.000474 | 0.0000945 | 0.0000804 | 1.27E-04 |
| PHBH-2 | S2-2 | 1005.1 | 436.821 | 5816.553 | 8063.349 | 0.00072 | 0.000461 | 0.0000925 | 0.0000781 | 1.32E-04 |
| PHBH-3 | S2-3 | 1042.766 | 434.321 | 5648.768 | 7830.752 | 0.000791 | 0.000507 | 0.0001011 | 0.0000858 | 1.35E-04 |
| PHBH-4 | S2-4 | 1219.271 | 488.423 | 6208.052 | 8606.074 | 0.000707 | 0.000452 | 0.0000902 | 0.0000765 | 1.38E-04 |
| PHBH-5 | S2-5 | 1152.798 | 445.521 | 5536.912 | 7675.688 | 0.000715 | 0.000458 | 0.0000915 | 0.0000774 | 1.41E-04 |
| PHBH-6 | S2-6 | 1231.672 | 460.481 | 5598.433 | 7760.973 | 0.000779 | 0.000499 | 0.0000991 | 0.0000843 | 1.43E-04 |
| PHBH-7 | S2-7 | 1413.122 | 512.323 | 6096.196 | 8451.011 | 0.000622 | 0.000399 | 0.0000794 | 0.0000673 | 1.46E-04 |
| PHBH-8 | S2-8 | 1185.325 | 417.618 | 4865.771 | 6745.301 | 0.000779 | 0.0005 | 0.0000993 | 0.0000843 | 1.49E-04 |
| PHBH-9 | S2-9 | 1557.002 | 534.123 | 6096.196 | 8451.01 | 0.000716 | 0.00046 | 0.0000911 | 0.0000773 | 1.52E-04 |
| PHBH-10 | S2-10 | 1494.442 | 500.021 | 5592.84 | 7753.22 | 0.000788 | 0.000506 | 0.0001002 | 0.000085 | 1.54E-04 |
| PHBH-11 | S2-11 | 1716.486 | 561.023 | 6152.124 | 8528.542 | 0.000611 | 0.0005 | 0.0000889 | 0.0000799 | 1.57E-04 |
| PHBH-12 | S2-12 | 320.016 | 7599.36 | 366.66 | 7975.64 | 0.000561 | 0.0005 | 0.0000889 | 0.0000799 | 1.60E-04 |
| PHBH-13 | S2-13 | 3129.21 | 1916.625 | 546.36 | 8722.68 | 0.000623 | 0.000555 | 0.0000987 | 0.0000887 | 2.87E-04 |
| PHBH-14 | S2-14 | 355.218 | 8435.29 | 333.3 | 8880.022 | 0.00111 | 0.000612 | 0.000421 | 0.000101 | 2.42E-04 |
| PHBH-15 | S2-15 | 2212 | 3221 | 2932 | 7987.11 | 0.001121 | 0.000619 | 0.0004252 | 0.000101 | 2.86E-04 |
| PES-1 | S2-16 | 2840.12 | 3253.21 | 2153.32 | 8167.981 | 0.000928 | 0.000532 | 0.000412 | 0.0000988 | 3.96E-04 |
| PES-2 | S2-17 | 1116.105 | 309.09 | 1478.12 | 7088.64 | 0.00091 | 0.000522 | 0.000391 | 0.0000978 | 4.19E-04 |
| PBS-1 | S2-18 | 1123.183 | 302.908 | 1379.958 | 6907.667 | 0.000939 | 0.000539 | 0.0003727 | 0.000102 | 2.51E-04 |
| PBS-2 | S2-19 | 1187.87 | 312.181 | 1351.501 | 7078.726 | 0.000838 | 0.000482 | 0.0003051 | 0.0000918 | 2.52E-04 |
| PBS-3 | S2-20 | 1085.495 | 278.181 | 1141.308 | 6271.776 | 0.001015 | 0.000584 | 0.0003368 | 0.0001123 | 2.50E-04 |
| PBS-4 | S2-21 | 1347.354 | 336.908 | 1305.951 | 7552.218 | 0.000923 | 0.000532 | 0.0002762 | 0.000103 | 2.49E-04 |
| PBS-5 | S2-22 | 1253.444 | 305.999 | 1116.839 | 6819.754 | 0.000933 | 0.000538 | 0.000249 | 0.000105 | 2.48E-04 |
| PBS-6 | S2-23 | 1296.105 | 309.09 | 1058.12 | 6848.64 | 0.000953 | 0.00055 | 0.0002234 | 0.0001081 | 2.48E-04 |
| PBS-7 | S2-24 | 1352.627 | 315.272 | 1007.882 | 6944.813 | 0.001021 | 0.00055 | 0.0003286 | 0.0001051 | 2.48E-04 |
| PBS-8 | S2-25 | 1139.288 | 318.363 | 1522.464 | 7301.299 | 0.00101 | 0.000606 | 0.000403 | 0.0001232 | 2.48E-04 |
| PBS-9 | S2-26 | 4446.131 | 2820.946 | 3862.644 | 6789.644 | 0.00105 | 0.000624 | 0.000415 | 0.0001269 | 2.46E-04 |
| PBAT-1 | S2-27 | 4598.994 | 2800.737 | 3977.376 | 6887.317 | 0.00101 | 0.000606 | 0.000403 | 0.0001232 | 4.75E-04 |
| PBAT-2 | S2-28 | 4486.531 | 2618.946 | 3862.644 | 6587.644 | 0.00099 | 0.000594 | 0.000395 | 0.0001208 | 4.79E-04 |
| PBAT-3 | S2-29 | 4417.489 | 2468.086 | 3786.156 | 6358.196 | 0.00109 | 0.000654 | 0.0004349 | 0.000133 | 4.79E-04 |
| PBAT-4 | S2-30 | 4885.5 | 2608.387 | 4168.596 | 6891.438 | 0.00101 | 0.000606 | 0.000403 | 0.0001232 | 4.81E-04 |
| PBAT-5 | S2-31 | 4547.131 | 2315.946 | 3862.644 | 6284.644 | 0.00098 | 0.000589 | 0.000391 | 0.0001196 | 4.83E-04 |
| PBAT6-6 | S2-32 | 4431.668 | 2149.156 | 3747.912 | 5999.972 | 0.00091 | 0.000547 | 0.0003631 | 0.0001119 | 4.85E-04 |
| PBAT-7 | S2-33 | 4133.32 | 1904.645 | 3480.204 | 5480.402 | 0.00106 | 0.000637 | 0.0004229 | 0.0001293 | 4.87E-04 |
| PBAT-8 | S2-34 | 4581.437 | 2854.597 | 4159.864 | 7231.765 | 0.00102 | 0.000619 | 0.0004333 | 0.000102 | 4.89E-04 |
| PBAT-9 | S2-35 | 6587.341 | 5384.083 | 4240.948 | 5858.02 | 0.000947 | 0.000543 | 0.0004202 | 0.0001008 | 4.69E-04 |
| PBSA-1 | S2-36 | 1015.529 | 1952.051 | 1366.989 | 3190.968 | 0.000975 | 0.000547 | 0.0003927 | 0.0001616 | 5.61E-04 |
| PBSA-2 | S2-37 | 1164.261 | 2344.956 | 1113.356 | 2103.513 | 0.001 | 0.000522 | 0.0003874 | 0.0001512 | 3.80E-04 |
| PBSA-3 | S2-38 | 1273.427 | 2004.422 | 1298.958 | 2646.824 | 0.000911 | 0.000592 | 0.000309 | 0.000101 | 4.44E-04 |
| PBSA-4 | S2-39 | 150.013 | 4250.4 | 1674.12 | 3380.36 | 0.000987 | 0.000537 | 0.0005171 | 0.0001101 | 4.46E-04 |
| PBSA-5 | S2-40 | 1284.877 | 2022.444 | 1653.213 | 3161.724 | 0.000933 | 0.000537 | 0.0002793 | 0.0001041 | 3.71E-04 |
| PBSA-6 | S2-41 | 1311.663 | 3067.178 | 1793.998 | 3747.59 | 0.001031 | 0.000568 | 0.0004573 | 0.0001121 | 4.34E-04 |
| PBSA-7 | S2-42 | 1102.902 | 2192.738 | 1135.236 | 2422.486 | 0.001071 | 0.000572 | 0.0003152 | 0.0001877 | 3.79E-04 |
| PBSA-8 | S2-43 | 8147.485 | 9057.374 | 4578.209 | 7341.634 | 0.00101 | 0.000523 | 0.0002818 | 0.0001061 | 4.17E-04 |
| PBSA-9 | S2-44 | 7955.982 | 23936.6 | 4533.365 | 7541.125 | 0.001061 | 0.000576 | 0.0003286 | 0.0001051 | 5.64E-04 |
| PBSA-10 | S2-45 | 7335.784 | 2025.248 | 3611.468 | 6176.086 | 0.001112 | 0.000654 | 0.0004349 | 0.000133 | 5.10E-04 |
| PBSA-11 | S2-46 | 8848.326 | 9700.758 | 4674.399 | 7736.471 | 0.00104 | 0.000593 | 0.0003727 | 0.0001444 | 5.45E-04 |
| PBSA-12 | S2-47 | 8229.207 | 9008.976 | 4230.324 | 7067.657 | 0.001061 | 0.000585 | 0.0003458 | 0.0001663 | 5.71E-04 |
| PBSA-13 | S2-48 | 8341.285 | 9118.574 | 4170.209 | 7035.634 | 0.001134 | 0.000606 | 0.0003337 | 0.0001987 | 5.73E-04 |
| PBSA-14 | S2-49 | 8864.348 | 9676.56 | 4307.515 | 7341.494 | 0.00106 | 0.000548 | 0.000279 | 0.000205 | 5.75E-04 |
| PBSA-15 | S2-50 | 8237.73 | 8979.778 | 3888.44 | 6697.68 | 0.001166 | 0.000583 | 0.0002714 | 0.0002453 | 5.78E-04 |
| PBSA-16 | S2-51 | 9011.826 | 9809.758 | 4129.4 | 7191.471 | 0.001296 | 0.000626 | 0.0002628 | 0.0002952 | 5.80E-04 |
| PBSA-17 | S2-52 | 9957.276 | 10823.73 | 4426.128 | 7797.216 | 0.001132 | 0.000625 | 0.0004274 | 0.000104 | 5.83E-04 |
| PBSA-18 | S2-53 | 7854.326 | 7411.616 | 1780.466 | 12035.63 | 0.001144 | 0.000631 | 0.0004326 | 0.000104 | 5.86E-04 |
| PCL-1 | S2-54 | 7972.53 | 7484.279 | 1767.022 | 11947.63 | 0.001121 | 0.000619 | 0.0004252 | 0.000101 | 5.24E-04 |
| PCL-2 | S2-55 | 7868.223 | 7278.353 | 1803.411 | 11614.64 | 0.001111 | 0.000613 | 0.000422 | 0.0000991 | 5.28E-04 |
| PCL-3 | S2-56 | 7810.32 | 7266.29 | 1725.555 | 11299.64 | 0.001154 | 0.000638 | 0.0004399 | 0.000102 | 5.30E-04 |
| PCL-4 | S2-57 | 8169.533 | 7525.742 | 1815.377 | 11595.63 | 0.00109 | 0.000601 | 0.0004155 | 0.0000952 | 5.33E-04 |
| PCL-5 | S2-58 | 7745.254 | 7085.684 | 1711.624 | 10769.85 | 0.00101 | 0.000558 | 0.0003868 | 0.0000875 | 5.36E-04 |
| PCL-6 | S2-59 | 7253.901 | 6574.104 | 1590.275 | 9854.972 | 0.001121 | 0.000619 | 0.0004303 | 0.0000961 | 5.39E-04 |
| PCL-7 | S2-60 | 8150.013 | 7290.473 | 1766.041 | 10776.34 | 0.000877 | 0.000484 | 0.0003373 | 0.0000743 | 5.43E-04 |
| PCL-8 | S2-61 | 6483.783 | 5697.709 | 1382.148 | 8302.616 | 0.001236 | 0.000631 | 0.000411 | 0.0001257 | 5.47E-04 |
| PCL-9 | S2-62 | 5377.857 | 7725 | 2362.861 | 7964.413 | 0.001 | 0.0006 | 0.000399 | 0.000112 | 5.52E-04 |
| PCL-10 | S2-63 | 5409 | 7230.03 | 2364.775 | 8442.22 | 0.00111 | 0.000613 | 0.000419 | 0.000102 | 5.59E-04 |
| PCL-11 | S2-64 | 5132.11 | 4824.46 | 2677.85 | 9975.44 | 0.001233 | 0.000559 | 0.0004034 | 0.0001233 | 4.96E-04 |
| PCL-12 | S2-65 | 5491.873 | 4688.634 | 2399.421 | 8312.887 | 0.00109 | 0.000654 | 0.0004349 | 0.000133 | 4.78E-04 |
| PCL-13 | S2-66 | 5315.069 | 4410.369 | 2495.43 | 10875.76 | 0.001101 | 0.000619 | 0.0004232 | 0.000103 | 5.39E-04 |
| PCL-14 | S2-67 | 12454.64 | 9790.511 | 2117.889 | 16766.2 | 0.001121 | 0.000619 | 0.0004232 | 0.000103 | 4.45E-04 |
| PCL-15 | S2-68 | 5273.432 | 7575 | 1212.364 | 16261.02 | 0.000885 | 0.000489 | 0.000292 | 0.0001039 | 5.40E-04 |
| PCL16 | S2-69 | 15789.87 | 16953.61 | 1270.374 | 10019.97 | 0.001121 | 0.000619 | 0.0004232 | 0.000103 | 4.18E-04 |
| PCL-17 | S2-70 | 7640.973 | 7439.953 | 1763.011 | 12927.64 | 0.001121 | 0.000619 | 0.0004232 | 0.000103 | 4.66E-04 |
| PCL-18 | S2-71 | 909.45 | 414.12 | 5648.77 | 7830.75 | 0.000742 | 0.000474 | 0.0000945 | 0.0000804 | 5.07E-04 |

## **Table S3****.** Meta-information of thermophysical properties, elements, and functional groups used for MBA.

Thermophysical properties (melting temperature, *T*_m_; Thermal decomposition temperature, *T*_d_; glass transition temperature, *T*_g_) of the polymer are shown. Aroma.: aromaticity, more 4 carbons: linearly connected methylenes > 4 carbons, oxygen: oxygen containing. Y / N is with or without measurement data or unknown.

| Sample ID | *T*_m_(℃) | *T*_g_(℃) | *T*_d_(℃) | more 4 carbons | oxygen | aroma. | Polymer name |
| --- | --- | --- | --- | --- | --- | --- | --- |
| 1 | 115 | 114 | 288 | N | Y | N | PHBH |
| 2 | N | N | 378 | N | Y | N | PES |
| 3 | 98 | N | 396 | N | Y | N | PES |
| 4 | 96 | N | 402 | N | Y | N | PES |
| 5 | 113 | 112 | 387 | Y | Y | N | PBS |
| 6 | 119 | N | 326 | Y | Y | N | PBS |
| 7 | 111 | 108 | 396 | Y | Y | Y | PBAT |
| 8 | 113 | 110 | 346 | Y | Y | Y | PBAT |
| 9 | 96 | 112 | 321 | Y | Y | N | PBSA |
| 10 | 94 | 91 | 306 | Y | Y | N | PBSA |
| 11 | 94 | 89 | 319 | Y | Y | N | PBSA |
| 12 | 114 | 112 | 319 | Y | Y | N | PBSA |
| 13 | 70 | 60 | 322 | Y | Y | N | PCL |
| 14 | 67 | 69 | 404 | Y | Y | N | PCL |
| 15 | 67 | 65 | 328 | Y | Y | N | PCL |
| 16 | 63 | N | N | Y | Y | N | PCL |
| 17 | 64 | N | N | Y | Y | N | PCL |
| 18 | 54 | N | 397 | Y | Y | N | PCL |
| 19 | 59 | 61.5 | 394 | Y | Y | N | PCL |
| 20 | 57 | 60.6 | 396 | Y | Y | N | PCL |
| 21 | 246 | 248 | 427 | N | Y | Y | PET |
| 22 | 248 | 248 | 424 | N | Y | Y | PET |
| 23 | 257 | 255 | 414 | N | Y | Y | PET |
| 24 | 259 | 259 | 417 | N | Y | Y | PET |
| 25 | 254 | 253 | 420 | N | Y | Y | PET |
| 26 | 120 | 121 | 472 | N | N | N | PE |
| 27 | 132 | N | 379 | N | N | N | PE |
| 28 | 113 | 109 | 460 | N | N | N | PE |
| 29 | 124 | 123 | 471 | N | N | N | PE |
| 30 | 129 | 127 | 481 | N | N | N | PE |
| 31 | 131 | 127 | 480 | N | N | N | PE |
| 32 | 193 | N | 382 | Y | Y | N | nylon |
| 33 | 212 | N | 383 | Y | Y | N | nylon |
| 34 | 239 | N | 367 | Y | Y | N | nylon |
| 35 | 152 | 150 | 304 | N | Y | N | PLA |
| 36 | 149 | 149 | 355 | N | Y | N | PLA |
| 37 | 151 | 148 | 352 | N | Y | N | PLA |
| 38 | 167 | N | 349 | N | Y | N | PLA |
| 39 | 161 | 159 | 327 | N | Y | N | PLA |
| 40 | 168 | 166 | 352 | N | Y | N | PLA |
| 41 | 169 | 170 | 357 | N | Y | N | PLA |
| 42 | 169 | 169 | 357 | N | Y | N | PLA |
| 43 | 168 | 166 | 355 | N | Y | N | PLA |
| 44 | 170 | 171 | 356 | N | Y | N | PLA |
| 45 | 146 | 150 | 356 | N | Y | N | PLA |
| 46 | 169 | 168 | 349 | N | Y | N | PLA |
| 47 | 162 | 162 | 358 | N | Y | N | PLA |
| 48 | 171 | 170 | 339 | N | Y | N | PLA |
| 49 | 162 | 161 | 405 | N | Y | N | PLA |
| 50 | 166 | 158 | 342 | N | Y | N | PLA |
| 51 | 169 | 168 | 303 | N | Y | N | PLA |
| 52 | 151 | 151 | 358 | N | Y | N | PLA |
| 53 | 163 | 162 | 356 | N | Y | N | PLA |
| 54 | 164 | 171 | 354 | N | Y | N | PLA |
| 55 | 164 | 162 | 354 | N | Y | N | PLA |
| 56 | 189 | 191 | 356 | N | Y | N | PLA |
| 57 | 150 | N | 317 | N | Y | N | PLA |
| 58 | 179 | N | N | N | Y | N | PLA |
| 59 | 150 | N | 357 | N | Y | N | PLA |
| 60 | 258 | N | 276 | N | Y | N | PLA |
| 61 | 130 | N | 454 | N | N | N | PP, PE |
| 62 | 167 | 167 | 455 | N | N | N | PP |
| 63 | 168 | 169 | 452 | N | N | N | PP |
| 64 | 144 | 143 | 449 | N | N | N | PP |
| 65 | 272 | 268 | 443 | N | Y | Y | PEN |
| 66 | N | N | 415 | N | N | Y | PS |
| 67 | 307 | N | 408 | N | N | Y | PS |
| 68 | 175 | 174 | 288 | N | Y | N | PA |
| 69 | 163 | 162 | 292 | N | N | N | PVDF |
| 70 | 190 | 162 | 252 | N | N | N | PVDC |
| 71 | N | N | 298 | N | N | N | PVC |
| 72 | 221 | 217 | 310 | N | Y | N | PGA |
| 73 | N | N | 412 | N | Y | Y | ABS |
| 74 | N | N | 155 | N | Y | N | PVA |
| 75 | 178 | 179 | 302 | N | Y | N | PVA |
| 76 | 129 | 128 | 477 | N | N | N | PE+10%BioPE |
| 77 | 115 | 112 | 356 | Y | Y | Y | PBS(A)/T |
| 78 | 112 | 110 | 392 | Y | Y | Y | PBS-PLA_copolymer |
